# S-palmitoylation and sterol interactions mediate antiviral specificity of IFITM isoforms

**DOI:** 10.21203/rs.3.rs-1179000/v1

**Published:** 2021-12-29

**Authors:** Tandrila Das, Xinglin Yang, Hwayoung Lee, Emma Garst, Estefania Valencia, Kartik Chandran, Wonpil Im, Howard Hang

**Affiliations:** Scripps Research; Scripps Research; Lehigh University; The Rockefeller University; Albert Einstein College of Medicine; Department of Microbiology and Immunology, Albert Einstein College of Medicine, Bronx, New York; Lehigh University; Scripps

## Abstract

Interferon-induced transmembrane proteins (IFITM1, 2 and 3) are important antiviral proteins that are active against many viruses, including influenza A virus (IAV), dengue virus (DENV), Ebola virus (EBOV), Zika virus (ZIKV) and severe acute respiratory syndrome coronavirus (SARS-CoV). IFITMs exhibit isoform-specific activity, but their distinct mechanisms of action and regulation are unclear. Since *S*-palmitoylation and cholesterol homeostasis are crucial for viral infections, we investigated IFITM interactions with cholesterol by molecular dynamic stimulations, nuclear magnetic resonance analysis *in vitro* and photoaffinity crosslinking in mammalian cells. These studies suggest that cholesterol can alter the conformation of IFITMs in membrane bilayers and directly interact with *S*-palmitoylated IFITMs in cells. Notably, we discovered that the *S*-palmitoylation levels regulate differential IFITM isoform interactions with cholesterol in mammalian cells and specificity of antiviral activity towards IAV, SARS-CoV-2 and EBOV. Our studies suggest that modulation of IFITM *S*-palmitoylation levels and cholesterol interaction may influence host susceptibility to different viruses.

## Introduction

Interferons (IFNs) provide an important host response to infection and induce the expression of many genes that protect against diverse pathogens^1^. Amongst the hundreds of IFN-stimulated genes (ISGs), interferon-induced transmembrane proteins (IFITMs) have emerged as crucial IFN effectors that prevent viral infection of host cells^2^. In particular, IFITM1, 2 and 3 have been shown to restrict infection by many enveloped viruses including IAV, DENV, EBOV, ZIKV, SARS-CoV, hepatitis C virus (HCV), human immunodeficiency virus (HIV) and others in mammalian cell lines^3–5^ and mouse models^6,7^. Notably, single nucleotide polymorphisms (SNPs) in humans, such as rs12252-C, which encodes a truncated form of IFITM3, have been associated with severe H1N1 IAV infection^7^. Also, IFITM3 SNP rs34481144, which results in lower mRNA expression, has been correlated with reduced CD8+ T cells in patient airways and increased IAV infection severity^8^. Moreover, IFITM3 SNP rs12252-C has been identified as a risk factor for severity of SARS-CoV-2 infection amongst COVID-19 hospitalized patients in two independent studies^9,10^. IFITMs are also expressed in embryonic stem cells independent of IFN stimulation and are important for their resistance to viral infections^11^. IFITMs reduce susceptibility of placental trophoblasts to viral infections and inhibit trophoblast cell fusion, an essential process for fetal development mediated by syncytin, a retroviral envelope-like protein^12,13^. These significant studies in cellular and animal models as well as preliminary clinical observations highlight an important function for IFITM3 in host immunity against viral infections. Beyond antiviral immunity, IFITM3 has been shown to be critical in phosphoinositide 3-kinase (PI3K) signal amplification for rapid expansion of B cells with high affinity to antigen^14^. IFITM3 is also implicated in neuroinflammatory conditions as it regulates γ-secretase activity and amyloid-β production in patients with late-onset Alzheimer’s disease^15^. These studies highlight broader roles of IFITMs in host physiology and disease, which requires a more detailed understanding of their mechanisms of action and regulation.

**Figure 1:**
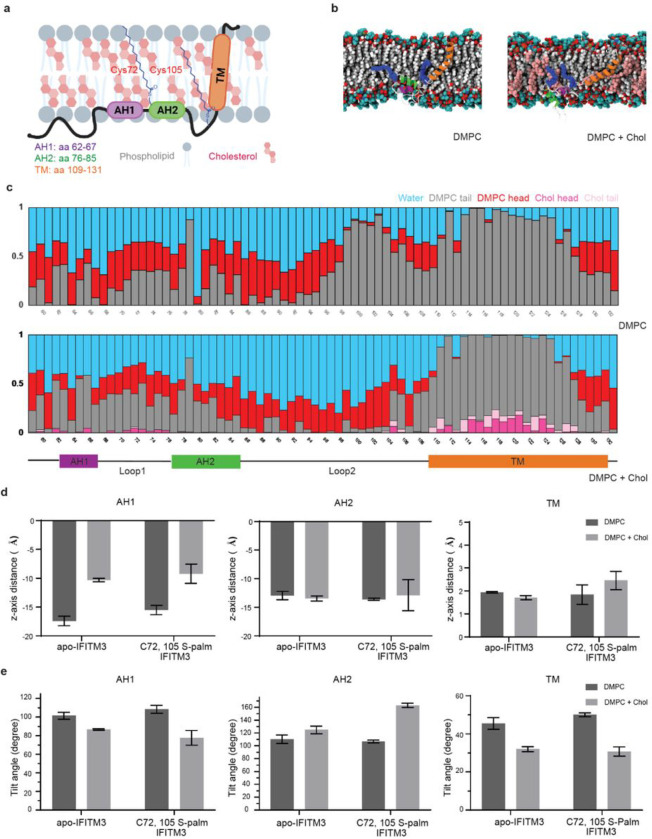
Molecular dynamics simulation of *S*-palmitoylated IFITM3 in cholesterol-containing membrane bilayer. **a**, *S*-palmitoylated IFITM3 in a membrane bilayer (created with Biorender.com). Key domains and post translational modifications are highlighted. Each secondary structure is colored differently, amphipathic helix 1 (AH1 from residue 62 to 67) in purple, amphipathic helix 2 (AH2 from residue 76 to 85) in green and transmembrane domain (TM from residue 109 to 131) in orange. **b**, Simulation snapshots of C72, 105 *S*-palm IFITM3 in DMPC (top) and DMPC + Chol membrane bilayers (bottom). **c**, Interaction frequency of each residue of C72, 105 *S*-palm IFITM3 in DMPC (top) and DMPC + Chol membrane bilayers (bottom) interacting with surrounding environment, DMPC lipid headgroups (red), DMPC lipid tails (gray), water (blue), cholesterol headgroup (magenta), and cholesterol tails (pink). Each graph shows the interaction frequency within 4 Å from each residue. **d**, The distance of the center of mass for each helical domain AH1, AH2 and TM to the membrane center (Z=0) for apo-IFITM3 and C72, 105 *S*-palm IFITM3 in DMPC or DMPC + Chol. Data represents mean and SE for triplicates. **e**, The averaged tilt angle of each helical domain AH1, AH2 and TM for apo-IFITM3 and C72, 105 *S*-palm IFITM3 in DMPC or DMPC + Chol. Averaged tilt angle was measured by calculating the angle between the principal axis of each helix and the membrane normal (Z-axis). Data represents mean and SE for triplicates.

IFITM1, 2 and 3 exhibit unique cellular properties and are highly regulated by posttranslational modifications^2,16^. All three IFITM isoforms show antiviral activity, but IFITM3 is the most active against many viruses^2,3,4^. In mammalian cells, IFITM2 and IFITM3 are distributed through early and late endosomes and lysosomes whereas IFITM1, which has a truncated N terminus localizes to the plasma membrane^2,4^. The cellular localization of IFITMs is an important determinant of their antiviral mechanism specificity, since enveloped viruses enter cells at different sites (for example, plasma membrane versus endocytic pathway) to deliver their genetic contents for replication in host cells. IFITMs are small type IV single-pass transmembrane proteins with an amphipathic helix that interacts with the inner membrane leaflet^17,18^ and are post-translationally modified by *S*-palmitoylation, phosphorylation, and ubiquitination (Fig. 1a). IFITM3 is *S*-palmitoylated at membrane-juxtaposed Cys residues (Cys71, 72 and 105)^19^, but Cys72 is the major site of fatty acid-modification^20,21^ and the most important for antiviral activity^20,22^. Recent molecular dynamic simulation and NMR studies show that fatty-acylation at Cys72 stabilizes IFITM3 amphipathic helix membrane interaction^23^. Notably, Cys72 is highly conserved across IFITM orthologs in mice, bats, and humans^20,24,25^. Live-cell imaging studies also show that Cys72 is essential for the trafficking of IFITM3-positive vesicles with incoming virus particles during infection^22^. In addition to fatty-acylation, Tyr20 phosphorylation regulates IFITM3 plasma membrane localization and endocytosis, whereas Lys ubiquitination at residues 24, 83, 88, and 104, and especially at Lys24, is important for IFITM3 trafficking and turnover in cells^26^.

The antiviral activity of IFITMs is also regulated by other protein and lipid interactions. For example, proteomic analysis of the *S*-palmitoylated IFITM3 interactome has identified many membrane-associated protein interaction partners^27–29^, including the p97/VCP ATPase that contributes to IFITM3 lysosomal turnover and antiviral activity^27,30,31^. Further, yeast two-hybrid screening revealed IFITM3 interaction with vesicle-membrane-protein-associated protein A (VAPA), which has been implicated in cholesterol accumulation in endolysosomal compartments and IFITM3 antiviral activity^31,32^. However, other studies indicate high cholesterol accumulation in these compartments by U18666A pretreatment or down-regulation of Niemann-Pick C1 (NPC1) does not inhibit IAV hemagglutinin-mediated viral membrane fusion^33^. Nonetheless, membrane cholesterol composition plays a critical role in viral infection by altering membrane mechanical properties and modulating membrane curvature during hemifusion or lipid mixing^34^. Furthermore, specific interactions of viral fusion proteins with cholesterol and other lipids are crucial for successful viral entry into cells^34,35^. Recent live-cell imaging studies show that IFITM3 colocalize with virus particles and traffics to lysosomes but does not inhibit membrane lipid mixing or hemifusion^22,36^. Despite its lack of effect on viral membrane hemifusion with endosomal membranes^22,33^, IFITM3 could potentially block fusion pore formation and release of viral genetic material in the cytosol. It has been suggested that the IFITM3 amphiphatic helix could modulate membrane curvature and stiffness to block fusion pore formation, a process that can be promoted by the presence of cholesterol in model membranes^17,37^. However, whether cholesterol directly binds IFITMs and affects their antiviral activity is unclear.

To investigate IFITM-cholesterol interactions, we employed *in silico,* structural and chemical biology approaches. Our molecular dynamics simulation suggests cholesterol alters the conformation of IFITM3 in membrane bilayers, which was supported by NMR studies of truncated IFITM3 protein constructs reconstituted in membrane bicelles. To characterize cholesterol interaction with IFITMs in mammalian cells, we synthesized a cholesterol photoaffinity reporter (x-alk-chol) and performed proteomic as well as targeted protein photocrosslinking studies. These studies demonstrated that x-alk-chol can photocrosslink IFITMs expressed in IFN-α-stimulated cells. Subsequent mutagenesis studies showed that *S*-palmitoylation and the CARC-cholesterol binding motif in the transmembrane domain of IFITM3 was important for x-alk-chol photoaffinity labeling and antiviral activity. Surprisingly, IFITM2 was photocrosslinked by x-alk-chol much less efficiently compared to IFITM3 even though these IFITM isoforms both contain the CARC-motif and are more than 80 percent identical at the protein level. Our subsequent analysis showed that the IFITM *S*-palmitoylation levels determined their x-alk-chol photocrosslinking and specificity of antiviral activity towards IAV, SARS-CoV-2 and EBOV. Collectively, our results suggest that cholesterol can modulate S-palmitoylated IFITM conformations in membrane bilayers, directly interact with IFITMs in mammalian cells and determine isoform-specific antiviral activity.

## Results

### Cholesterol alters IFITM3 conformations in membrane bilayers from molecular dynamics simulations.

To explore the effect of cholesterol on IFITMs in membrane bilayers, we performed molecular dynamics (MD) simulations of apo-IFITM3 and C72, C105 S-palmitoylated (palm) IFITM3 in a 1,2-dimyristoyl-*sn*-glycero-3-phosphocholine (DMPC) membrane bilayer with or without 20% cholesterol (Fig. 1b, Supplementary Fig. 1a, Supplementary table 1). We measured tilt angles with respect to the membrane normal (i.e., Z-axis), Z positions of the center of mass of each helix with respect to the bilayer center (i.e., Z=0), as well as the interaction patterns of each residue to quantify behaviors of each helical domain namely, amphipathic helix 1 (AH1, residues 62–67), amphipathic helix 2 (AH2, residues 76–85) and transmembrane domain (TM, residues 109–131). Each helical domain shows different behaviors on addition of cholesterol in DMPC membrane systems. However, we observed similar change in trends for the helices in both apo-IFITM3 and C72, 105 S-palm IFITM3. C72, 105 S-palm IFITM3, shows more AH1 and Loop2 (residues 86–108) interactions with DMPC membrane than apo-IFITM3 (Fig. 1b, Supplementary Fig. 1a, Supplementary table 1). This is consistent with our previous studies, which showed that *S*-palmitoylation at Cys72 increased IFITM3 AH1 interaction with DMPC membranes^23^. In the presence of cholesterol, both apo and C72, 105 *S*-palm IFITM3 AH1 shows further increase in membrane interactions as its tilt angles are closer to 90°, a completely horizontal orientation. AH1 has a tilt angle of 86.85° for apo-IFITM3 and 77.7° for C72, 105 *S*-palm IFITM3 in cholesterol containing systems versus 101.53° for apo-IFITM3 and 108.4° for C72, 105 *S*-palm IFITM3 in DMPC-only systems (Supplementary table 2). Additionally, AH1 domain was more deeply embedded into the membrane in the presence of cholesterol, thus making more frequent interactions with lipid tails (Fig. 1c, d, Supplementary Fig. 1b). AH2, on the other hand, responses differently to the presence of cholesterol in membrane in comparison to AH1. AH2 tilt angle shows values larger than 90°, meaning that it becomes more vertical in membrane system with cholesterol (Supplementary table 2). In addition, presence of cholesterol does not affect the position of AH2 along the membrane normal in contrast to AH1 (Fig. 1d). For TM domain, the tilt angle tends to decrease in cholesterol membrane system for both apo-IFITM3 and C72, 105 *S*-palm IFITM3 (Fig. 1e). Such tilt angle changes are known to occur to maximize a hydrophobic match between the lipid bilayer and the transmembrane domain^38^. Cholesterol is known to increase the thickness and order of a lipid bilayer^39^, as we observed for all cholesterol containing membrane systems (Supplementary Fig. 2). Interestingly, we observe significant changes in the interaction pattern for Loop2 in membrane with and without cholesterol for both apo-IFITM3 and C72, 105 *S*-palm IFITM3. Loop2 is a part of the highly conserved CD225 domain which contains a basic patch consisting of R85, R87, and K88, as well as a ^91^GxxxG^95^ motif implicated in IFITM3 oligomerization and antiviral activity^25,40^. We see increased hydrophobic interactions in Loop2 in the DMPC system, whereas the cholesterol containing membrane system has more hydrophilic (protein-water and protein-lipid headgroup) interactions (Supplementary Fig. 3–6). Since DMPC is a fully saturated lipid and is well-mixed with rigid cholesterol, it leads to a more ordered membrane phase. This makes it difficult for Loop 2 to insert into the membrane hydrophobic core and have hydrophobic interactions. Thus, cholesterol-induced changes in IFITM3-membrane interactions might in turn influence IFITM3 interactions with other lipids and proteins as well as its activity.

To structurally characterize IFITM3 in a cholesterol-containing bilayer, we employed a bicelle membrane bilayer system. However, in the presence of full length IFITM3, the short chain lipid, 1,2-dihexanoyl-*sn*-glycero-3-phosphatidylcholine (DHPC) could not solubilize DMPC liposomes to form membrane bicelle. We therefore designed several truncated constructs for bicelle reconstitution. IFITM3^89−133^, which excluded the amphipathic helices, expressed well after overnight induction, and were purified using His-affinity purification (Supplementary Fig. 7a,b). We characterized ^15^N-labeled IFITM3^89−133^ in bicelles with a protein to lipid ratio of 1:50 and final q value between 0.5 and 0.6 using 2D 1H-15N TROSY spectra (Supplementary Fig. 8). We observed subtle changes in some specific residues of the transmembrane domain though full assignment of the transmembrane region residues could not be performed due to low signal. This suggests specific structural changes in the IFITM3 transmembrane domain in the presence of cholesterol in the bicelle membrane system.

### Photoaffinity labeling identifies IFITM3 as a cholesterol binding immune associated protein.

**Figure 2:**
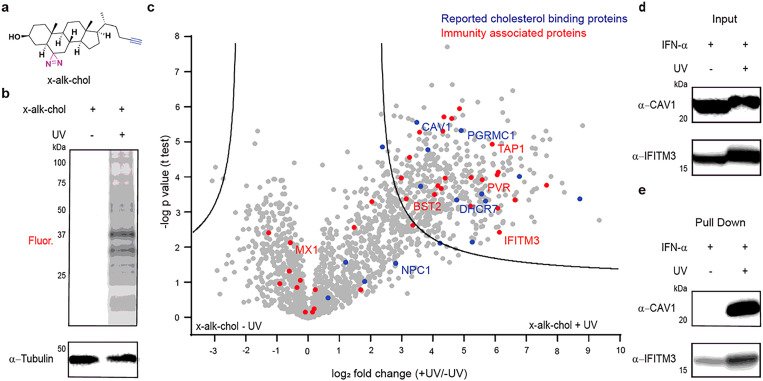
Cholesterol photoaffinity reporter probes cholesterol binding proteins in interferon stimulated HeLa cells. **a,** Structure of cholesterol photoaffinity reporter, x-alk-chol. **b,** In-gel fluorescence profiling of x-alk-chol crosslinked HeLa cell proteins shows UV dependent x-alk-chol labeling of many proteins in HeLa cell proteome. **c,** Volcano plot showing x-alk-chol crosslinked proteins in interferon (IFN-α) stimulated HeLa cells. Volcano plot shows enrichment of many reported cholesterol binding proteins (in blue) and new cholesterol binding immunity associated proteins (in red) for UV treated sample. The x-axis is the difference of means between the UV treated and untreated samples and the y-axis is the log of the probability of that difference determined by the t-test. The minimum values for a valid protein are p<0.05 and a difference of means of 2 (FDR=0.0001, S0=1, n=3 replicates). **d,** Western blot analysis of x-alk-chol binding proteins CAV1 and IFITM3. Interferon (IFN-α) treated cells were incubated with x-alk-chol (10 μM) for 30 min and UV-irradiated for 5 min and after cell lysis, proteins were separated by SDS-PAGE. Western blot analysis shows slower migration rate for CAV1 and IFITM3 (hits from proteomics analysis in c) for UV treated sample suggesting x-alk-chol crosslinking. **e,** Western blot analysis of streptavidin-biotin pull down of x-alk-chol crosslinked proteins. Cell lysates from d was reacted with azide-biotin for streptavidin pull down of x-alk-chol crosslinked proteins. Further western blot analysis shows UV dependent pull down of endogenous CAV1 and IFITM3.

To investigate cholesterol binding of IFITMs in mammalian cells, we synthesized a bifunctional cholesterol analog (x-alk-chol) that has a diazirine in position 6 for crosslinking to interacting proteins on UV light exposure (Fig. 2a, Supplementary Fig. 9). This cholesterol photoaffinity probe (x-alk-chol) also has a terminal alkyne handle on the alkyl side chain for Cu^I^-catalyzed azide-alkyne cycloaddition (CuAAC) to azide-fluorophore for fluorescence detection or azide-biotin for affinity enrichment of interacting proteins (Fig. 2a). Such photoaffinity cholesterol probes have been used to study sterol-protein interactions in cells previously^41,42^. Maestro modeling of x-alk-chol showed similar molecular topology as cholesterol (Supplementary Fig. 10). To probe cholesterol-binding proteins in cells, HeLa cells were treated with x-alk-chol and irradiated with UV light for x-alk-chol crosslinking with interacting proteins. The cell lysate was then subjected to CuAAC with azide-rhodamine for in-gel fluorescence profiling of x-alk-chol crosslinked proteins (Supplementary Fig. 11a). Many proteins in HeLa cells were crosslinked with x-alk-chol in a UV-dependent and dose-dependent manner (Fig. 2b and Supplementary Fig. 11b). Cells delivered excess cholesterol following x-alk-chol treatment showed lower labeling suggesting cholesterol competition with x-alk-chol (Supplementary Fig. 11c).

To enrich and identify x-alk-chol labeled proteins in HeLa cells, we subjected the cell lysate to CuAAC with azide-biotin, affinity purified with streptavidin beads and subjected to on-bead protease digestion for mass spectrometric analysis (Supplementary Fig. 12a). A summary of the proteomics data revealed enrichment of 330 proteins in UV light irradiated samples including known cholesterol binding proteins like caveolin-1 (CAV1) and Niemann-Pick disease, type C1 (NPC1), among others (Supplementary Fig. 12a)^43,44^. Bioinformatic analysis of the enriched proteins suggest x-alk-chol labeling of endoplasmic reticulum (ER) and plasma membrane resident membrane proteins primarily (Supplementary Fig. 12b). Many of the high confidence x-alk-chol labeled proteins, were previously identified in a proteome-labeling profile of trans-sterol probe (Supplementary Fig. 12c)^41^. To characterize cholesterol binding to IFITMs and other immune-associated proteins, we profiled x-alk-chol crosslinked proteins in IFN-α stimulated HeLa cells (Fig. 2c). In addition to the proteins identified in non-stimulated cells, we recovered IFITM3 and many other immune-associated proteins from the proteomic data (Fig. 2c). Western blot analysis of IFN-α stimulated and x-alk-chol treated HeLa cells showed a mobility shift for IFITM3 in UV-irradiated samples (Fig. 2d) resembling that seen with CAV1, a known cholesterol binding protein (Fig. 2d). To further confirm x-alk-chol photocrosslinking to IFITM3, the cell lysate was subjected to CuAAC with azide-biotin and affinity purified with streptavidin beads. Western blot analysis showed significant enrichment of IFITM3 and CAV1 in the UV-irradiated sample (Fig. 2e). Gene ontology analysis of the proteins enriched only in IFN-α stimulated cells identified many other membrane-associated proteins involved in lipid biosynthesis, metabolism and immunity (Supplementary Fig. 13a, b), which may warrant further investigation as many of these hits are important host factors involved in virus infection including SARS-CoV-2^45,46^. These results show that x-alk-chol can photocrosslink many known cholesterol-binding proteins as well as capture many immune-associated proteins including IFITM3.

### IFITM3-cholesterol interaction is important for antiviral activity.

**Figure 3:**
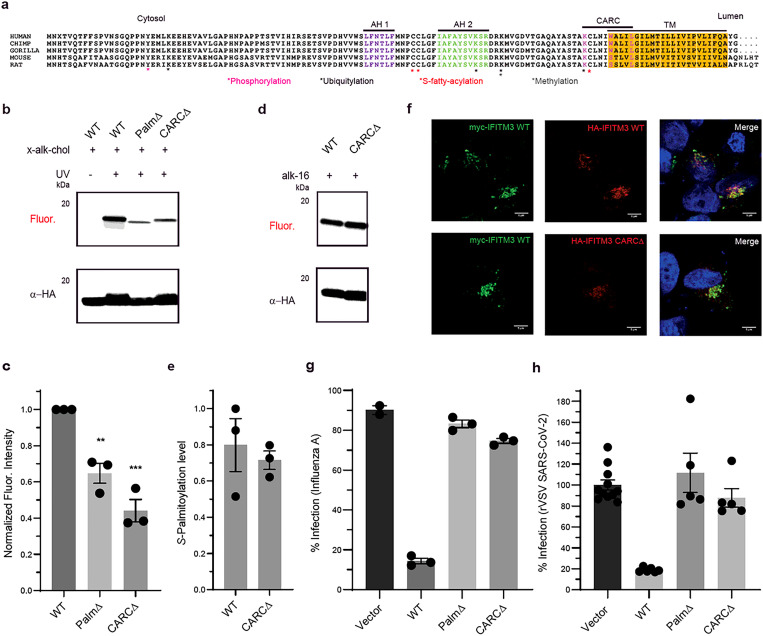
IFITM3 cholesterol binding regulates antiviral activity. **a,** Alignment, and topology of primate and rodentia IFITM3. Sequence alignment shows conserved cholesterol binding domain across primate IFITM3 homologues. The three residues (Lys, Trp and Leu) important for cholesterol binding are in pink. **b,** x-alk-chol labeling of overexpressed HA-tagged IFITM3 wildtype and cholesterol binding mutants. In-gel fluorescence profiling shows UV-dependent fluorescence signal for IFITM3 WT. IFITM3 PalmΔ and CARCΔ shows significantly lower fluorescence signal suggesting lower level of x-alk-chol labeling. Anti-HA blot shows IFITM3 expression for each construct. **c,** Quantitative analysis of x-alk-chol labeling of IFITM3 constructs in b. The fluorescence signal normalized to protein level was plotted for each construct. Data represents the mean and s.e.m. of three independent experiments. P values were determined by one-way anova with a Dunnett’s multiple comparisons test. **P<.01, ***P<.001. **d,** *S*-palmitoylation analysis of overexpressed HA-tagged IFITM3 WT and CARCΔ. In-gel fluorescence profiling shows similar metabolic labeling of IFITM3 WT and CARCΔ with alk-16 labeling. Anti-HA blot shows IFITM3 expression for each construct. **e,** Quantitative analysis of alk-16 labeling of IFITM3 constructs in d. The fluorescence signal normalized to protein level was plotted for each construct. Data represents the mean and s.e.m. of three independent experiments. **f,** Subcellular localization of IFITM3 WT and CARCΔ. Top row, localization of myc-IFITM3 WT (green) and HA-IFITM3 WT (red); bottom row, myc-IFITM3 WT (green) and HA-IFITM3 CARCΔ (red). **g,** Influenza A virus (IAV) infection of A549 IFITM1/2/3 KO cells stably expressing IFITM3 constructs. Virus nucleoprotein (NP) levels were examined by flow cytometry for percentage of infection analysis. Data represents mean and s.e.m. for triplicates. **h,** rVSV-SARS-CoV-2 S infection of A549 IFITM1/2/3 KO cells stably expressing IFITM3 constructs. Viral Infectivity was examined by counting the eGFP-positive cells using fluorescent microscopy. Data represents mean and s.e.m. for five independent experiments.

*S*-palmitoylated membrane proteins have been suggested to partition into cholesterol-rich liquid-ordered membrane microdomains^47,48^. IFITM3 is *S*-palmitoylated at Cys71, 72 and 105 (Fig. 3a). To validate IFITM3 cholesterol interaction, we performed x-alk-chol crosslinking studies with overexpressed HA tagged IFITM3 WT in HEK293T cells. In-gel fluorescence detection of anti-HA immunoprecipitated sample, shows UV-dependent x-alk-chol crosslinking of HA-IFITM3 (Fig. 3b). To identify the role of *S*-palmitoylation in IFITM3 cholesterol binding, we tested x-alk-chol labeling of IFITM3 PalmΔ construct, a Cys71, 72 and 105 to Ala triple mutant (Fig. 3b, c). IFITM3 PalmΔ shows significantly less x-alk-chol labeling. Bioinformatic analysis of IFITM3 orthologues from great apes and rodentia shows that IFITM3 has a putative cholesterol binding domain. IFITM3 in great apes have a cholesterol consensus domain CARC ^104^KCLNIWALIL^113^ (in pink) which lies N-terminus to the transmembrane domain (Fig. 3a). Many membrane proteins have CARC motifs or its mirror code CRAC motif to mediate interactions with cholesterol^49,50^. To investigate role of CARC motif in IFITM3-cholesterol interaction, we made a CARCΔ construct by replacing Lys104 with Ala and Trp109 with Ile to disrupt potential interaction with cholesterol hydroxyl group and sterol ring, respectively. We observe that x-alk-chol photocrosslinking efficiency of CARCΔ is significantly decreased (Fig. 3b, c). Thus, suggesting role of CARC motif in IFITM3-cholesterol interaction. IFITM3 Lys104 to Ala and Trp109 to Ile single mutants also show reduction in x-alk-chol photocrosslinking (Supplementary Fig. 14). IFITM3 single Cys71 and Cys72 to Ala mutants show similar x-alk-chol photocrosslinking as IFITM3 WT but Cys105 to Ala mutant shows some increase in x-alk-chol photocrosslinking. This may be due to less steric interactions at the CARC domain on mutation of Cys105 to Ala (Supplementary Fig. 15). Mouse IFITM3 does not have CARC domain conserved and shows lower levels of x-alk-chol photocrosslinking (Supplementary Fig. 16). *S*-palmitoylation levels of IFITM3 CARCΔ were analyzed by metabolic labeling with alk-16 labeling^19^. IFITM3 CARCΔ shows similar *S*-palmitoylation levels as IFITM3 WT (Fig. 3d, e). We also analyzed the subcellular localization of IFITM3 CARCΔ by co-expressing myc-tagged IFITM3 WT with HA-tagged IFITM3 CARCΔ. IFITM3 CARCΔ shows similar endolysosomal localization as IFITM3 WT (Fig. 3f, supplementary Fig. 17). These results suggest CARC domain of IFITM3 is crucial for its interaction with cholesterol but does not impact *S*-palmitoylation or subcellular localization.

To analyze the significance of IFITM3 interaction with cholesterol, we evaluated antiviral activity of IFITM3 cholesterol binding mutants against IAV and SARS-CoV-2. A549 IFITM1/2/3 KO - ACE2 cells stably expressing IFITM3 constructs were used for the infection studies (Supplementary Fig. 18). Cells expressing IFITM3 WT shows antiviral activity against IAV whereas loss of function construct IFITM3 PalmΔ has little or no activity^19^. Cells expressing IFITM3 CARCΔ construct shows significant loss of resistance to IAV infection (Fig. 3g, Supplementary Fig. 19). HEK293T cells expressing these IFITM3 constructs show similar trend in antiviral activity against IAV (Supplementary Fig. 20a, b). Next, we tested antiviral activity of these cell lines against a recombinant vesicular stomatitis virus bearing SARS-CoV-2 spike (rVSV-SARS-CoV-2 S)^51^ since recent studies show that SARS-CoV-2 spike protein interaction with cholesterol is important for virus entry and pathological syncytia formation^35^. However, the role of IFITMs in SARS-CoV-2 infection is unclear, since early studies show conflicting results for both inhibition of SARS-CoV-2 pseudotyped virus infection and SARS-CoV-2 spike protein mediated cell-cell fusion^35,52,53,54^. Furthermore, although overexpression of IFITMs restrict SARS-CoV-2 infection, endogenous levels of IFITMs was suggested to have a proviral effect^55,56^. Our A549 IFITM1/2/3 KO cells provides an excellent system to evaluate the activity of IFITMs in SARS-CoV-2 entry (Supplementary Fig. 18). Cells expressing IFITM3 WT shows antiviral activity against rVSV-SARS-CoV-2 S whereas IFITM3 PalmΔ or CARCΔ mutants have minimal or no antiviral activity (Fig. 3h). Thus, IFITM3-cholesterol interaction might play an important role in blocking virus fusion and release of genetic material in host cytosol during IAV and SARS-CoV-2 infection entry.

### IFITMs show isoform-specific x-alk-chol photocrosslinking and antiviral activity in cells.

**Figure 4:**
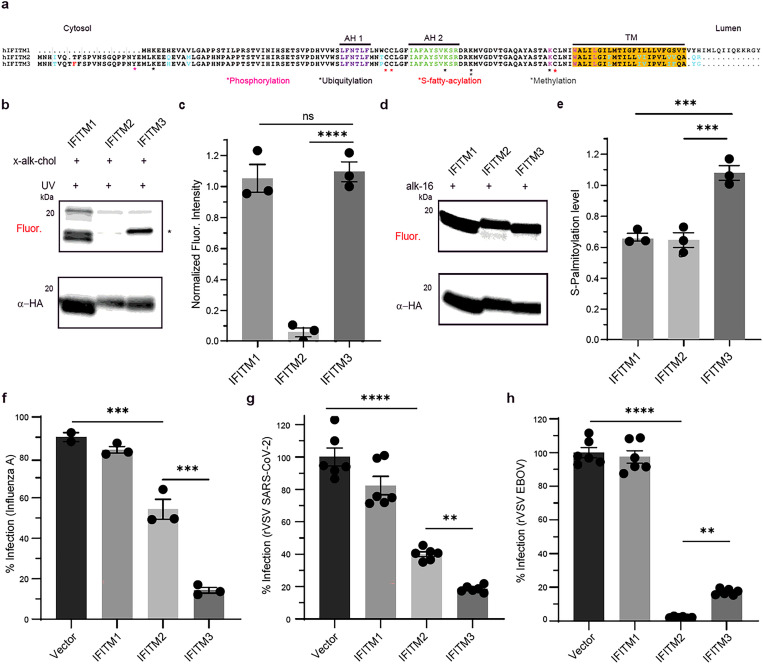
Cholesterol binding, *S*-palmitoylation and antiviral activity of IFITM isoforms. **a,** Alignment, and topology of interferon (IFN)-induced IFITM isoforms. The distinct residues in IFITM2 and IFITM3 are in cyan. IFITM3 has an additional Phe residue in red. **b,** x-alk-chol labeling of overexpressed HA-tagged IFITM isoforms and mutants. In-gel fluorescence profiling shows UV-dependent fluorescence signal for IFITMs. Anti-HA blot shows IFITM expression for each construct. **c,** Quantitative analysis of x-alk-chol labeling of IFITM constructs in b, d respectively. The fluorescence signal normalized to protein level was plotted for each construct. Data represents the mean and s.e.m. of three independent experiments. P values were determined by one-way anova with a Dunnett’s multiple comparisons test. 0.5<ns, ****P<.0001. **d,** *S*-palmitoylation analysis of overexpressed HA-tagged IFITM constructs. In-gel fluorescence profiling shows UV-dependent fluorescence signal for IFITMs. Anti-HA blot shows IFITM3 expression for each construct. **e,** Quantitative analysis of alk-16 labeling of IFITM3 constructs in d. The fluorescence signal normalized to protein level was plotted for each construct. Data represents the mean and s.e.m. of three independent experiments. P values were determined by one-way anova with a Dunnett’s multiple comparisons test. 0.5<ns, **P<.01, ***P<.001. **f,** Influenza A virus (IAV) infection of HEK293T and A549 IFITM1/2/3 KO cells expressing IFITM constructs. Cells were infected with IAV (MOI 10) for 6 h. Virus nucleoprotein (NP) levels were examined by flow cytometry using anti-NP staining for percentage of infection analysis. Data represents mean and s.e.m. for three independent experiments. Data represents the mean and s.e.m. of three independent experiments. P values were determined by one-way anova with a Tukey’s multiple comparisons test. ***P<.001. **g,** SARS-CoV-2 entry in A549 IFITM1/2/3 KO cells stably expressing IFITM isoforms. Cells were infected with rVSV-eGFP SARS-CoV-2 for 7 h. Viral Infectivity was examined by counting the eGFP-positive cells using fluorescent microscopy. Data represents mean and s.e.m. for five independent experiments. P values were determined by one-way anova with a Tukey’s multiple comparisons test. 0.5<ns, **P<.01, ****P<.0001. **h,** EBOV entry in A549 IFITM1/2/3 KO cells stably expressing IFITM isoforms. Cells were infected with rVSV-eGFP EBOV GP for 7 h. Viral Infectivity was examined by counting the eGFP-positive cells using fluorescent microscopy. Data represents mean and s.e.m. for five independent experiments. P values were determined by one-way anova with a Tukey’s multiple comparisons test. 0.5<ns, **P<.01, ****P<.0001.

We next analyzed cholesterol binding of human IFN-induced isoforms IFITM1, IFITM2 and IFITM3. All three IFITM isoforms have conserved Cys71, 72, 105 and cholesterol binding motif CARC (Fig. 4a). We used in-gel fluorescence profiling to evaluate photocrosslinking of IFITM isoforms with x-alk-chol. In-gel fluorescence detection and quantification normalized to protein levels showed x-alk-chol photocrosslinking to IFITM1 and IFITM3 but not with IFITM2 (Fig. 4b, c). Differential photocrosslinking IFITM2 and IFITM3 with x-alk-chol is surprising since sequence alignment of the isoforms show 83% sequence identity. These IFITM isoforms also showed similar subcellular localization by immunofluorescence confocal microscopy (Supplementary Fig. 21). However, the *S*-palmitoylation levels of IFITM2 and IFITM3 by alk-16 labeling correlated with x-alk-chol photocrosslinking (Fig. 4d, e). These results suggest IFITM2 and IFITM3 interaction with cholesterol maybe determined by their *S*-palmitoylation levels.

We then evaluated the antiviral activity of IFITM isoforms with IAV, SARS-CoV-2 and EBOV entry. IAV and SARS-CoV-2 use sialylated EGFR^57^ and ACE2^58^ as entry factor respectively whereas EBOV, another virus entering from late endosomes, has been shown to use NPC1 as an entry factor^59–61^. For these experiments, we stably expressed the IFITM isoforms in A549 IFITM1/2/3 KO - ACE2 cells (Supplementary Fig. 22a, b). Consistent with previous reports^62,63^, expression of plasma membrane resident IFITM1 did not inhibit infection by these viruses that enter through low pH endosomal compartments (Fig. 4f, g, h). In contrast, we found that IFITM3 exhibits greater antiviral activity against IAV and rVSV-SARS2 S whereas IFITM2 is more active against an rVSV bearing the EBOV spike glycoprotein, GP (rVSV-EBOV GP)^64^ (Fig. 4f, g, h). These results suggest differential interaction with cholesterol and *S*-palmitoylation may impact the antiviral activity of IFITM isoforms towards different viruses.

## Discussion

IFITMs have emerged as important IFN-induced effectors in host immunity against diverse viruses but require further mechanistic insight. Here, we explored IFITM-cholesterol interactions using *in silico,* structural and chemical biology approaches. Molecular dynamics simulation revealed potential effects of cholesterol on IFITM conformations and membrane interactions in lipid bilayers, which was supported in part by our NMR studies of IFITM3 transmembrane domain reconstituted in membrane bicelles. Our *in silico* studies also suggested that cholesterol may impact IFITM3 AH1 and Loop2 interactions with the membrane. This is particularly interesting since IFITM3 AH1 is important in inhibiting viral infection in cells^17,37^. Moreover, IFITM3 Loop2 contains motifs implicated in IFITM3 oligomerization and antiviral activity^25,40^. Further NMR studies of truncated IFITM3 in reconstituted bicelle membrane systems suggest specific structural changes in IFITM3 transmembrane domain in the presence of cholesterol. These cholesterol-induced changes in IFITM3 conformation in membranes as implicated in the *in silico* and NMR studies might in turn influence its interactions with other proteins as well as antiviral activity.

To complement the *in silico* and *in vitro* studies, we employed photoaffinity crosslinking to investigate direct cholesterol-protein interactions in cells. Our proteomic analysis of cholesterol photoaffinity crosslinking revealed many candidate sterol-interacting proteins, including endogenously expressed IFITM3 in interferon (IFN-α)-stimulated cells. Further targeted crosslinking studies identified a cholesterol binding motif (CARC) adjacent to the transmembrane domain of IFITM3. Infection studies with IFITM3-CARC cholesterol binding mutants suggest IFITM3-cholesterol interaction might play an important role in blocking virus entry into host cells mediated by the IAV and SARS-CoV-2 spike glycoproteins. This is in agreement with recent *in vitro* work, which shows cone-shaped lipids like cholesterol facilitate IFITM3-induced membrane curvature to inhibit viral fusion with host membranes^37^. Moreover, we show that *S*-palmitoylation of IFITM3 is crucial for cholesterol photoaffinity crosslinking in cells. Additional studies with different IFITM isoforms led to the surprising discovery that IFITM2 exhibits significantly less x-alk-chol crosslinking than IFITM3, even though these isoforms contained the conserved cholesterol binding CARC domain.

Further analysis by metabolic labeling revealed that *S*-palmitoylation level of IFITM2 is significantly less than IFITM3, which may explain the differential x-alk-chol crosslinking of the IFITM isoforms. Interestingly, infection studies in A549 cell lines shows IFITM3 has greater antiviral activity against IAV and SARS-CoV-2 infection, whereas IFITM2 is more active against EBOV, that enter host cells through interactions with NPC1 in late endosomes^59–61,65^. This is in agreement with previous observations that IFITM3 restricts viruses like IAV or flaviviruses more efficiently than cathepsin-dependent viruses like EBOV^59–61,65^. Interestingly, amphotericin B, which can bind cholesterol, has been reported to inhibit IFITM3 restriction of IAV but not EBOV,^62^ suggesting differential engagement of cholesterol-rich membranes or endosomes by IFITMs may impact selective inhibition of IAV versus EBOV infection. Our results now provide direct evidence for this possibility, as we show higher levels of *S*-palmitoylation enhance IFITM3 interaction with cholesterol and inhibits viruses like IAV and SARS-CoV-2, whereas IFITM2 exhibits lower *S*-palmitoylation levels and cholesterol interactions but more effectively inhibits EBOV, which may engage cellular membranes with less cholesterol that contain NPC1 transporter. Therefore, while both IFITM2 and IFITM3 are present in the endolysosomal pathway, their differential *S*-palmitoylation levels and cholesterol interactions may provide specificity against viruses that enter through different endosomal trafficking routes and membrane compartments. Therefore, it will be interesting to investigate the cellular and biochemical mechanisms that regulate differential palmitoylation and depalmitoylation of IFITM isoforms.^66,67^ Our study highlights how direct cholesterol photoaffinity labeling in cells can reveal unpredicted covalent and non-covalent lipid regulation of IFN effectors that maybe harnessed to modulate their activity and/or specificity towards different viruses in the future.

## Supplementary Material

Supplement 1

Supplement 2

## Data Availability

The data and materials that support the findings of this study are available from the corresponding author upon reasonable request.
