# Supplementary material for: S-palmitoylation and sterol interactions mediate antiviral specificity of IFITM isoforms
Source: Res Sq. 2021 Dec 29:rs.3.rs-1179000. Preprint. [Version 1] doi: 10.21203/rs.3.rs-1179000/v1 (PMC8722608; doi:10.21203/rs.3.rs-1179000/v1)
Supplement: Supplement 1 [file 01398e8486ce8a9b3b97fe0c.docx]

**Supplementary Information**

***S-palmitoylation and sterol interactions mediate antiviral specificity of IFITM isoforms***

Tandrila Das^1,2,3^, Xinglin Yang^3^, Hwayoung Lee^4^, Emma H. Garst^1,2^, Estefania Valencia^5^, Kartik Chandran^5^, Wonpil Im^4^, Howard C. Hang^3,6^*

^1^Laboratory of Chemical Biology and Microbial Pathogenesis, The Rockefeller University, New York, NY 10065, United States.

^2^Tri-Institutional Ph.D. Program in Chemical Biology, New York, NY 10065, United States.

^3^Department of Immunology and Microbiology, Scripps Research, La Jolla, CA 92037, United States.

^4^Department of Biological Sciences, Chemistry, and Bioengineering, Lehigh University, Bethlehem, PA 18015, United States.

^5^Department of Microbiology and Immunology, Albert Einstein College of Medicine, Bronx, NY, United States.

^6^Department of Chemistry, Scripps Research, La Jolla, CA 92037, United States.

*corresponding author

| **Table of contents** | **Page** |
| --- | --- |
| **I. Supplementary Figure 1-20** | **S2** |
| **II. Table S1-S2** | **S17** |
| **III. Materials and Methods** | **S18** |
| **IV. NMR spectrum of x-alk-chol** | **S22** |
| **V. References** | **S23** |

**
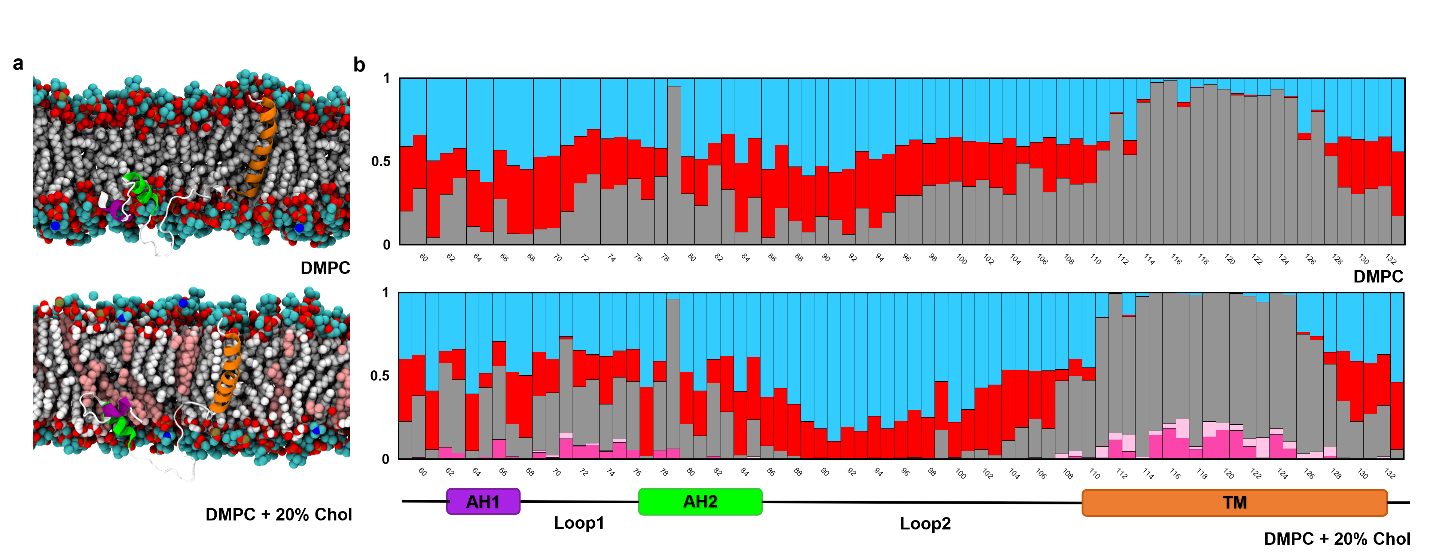
Supplementary Figure 1. a,** Simulation snapshots of apo-IFITM3 in DMPC (top) and DMPC + Chol membrane bilayers (bottom). Each secondary structure is colored differently: amphipathic helix 1 (AH1 from residue 62 to 67) in purple, amphipathic helix 2 (AH2 from residue 76 to 85) in green and transmembrane domain (TM from residue 109 to 131) in orange. **b,** Interaction frequency of each residue of C72, 105 S-palmitoylated IFITM3 in DMPC (top) and DMPC + Chol membrane bilayers (bottom) interacting with surrounding environment, DMPC lipid headgroup (red), DMPC lipid tails (gray), water (blue), cholesterol headgroup (magenta), and cholesterol tails (pink). Each graph shows the interaction frequency within 4 Å from each residue.

**Supplementary Figure 2.** Membrane thickness of each simulation system.


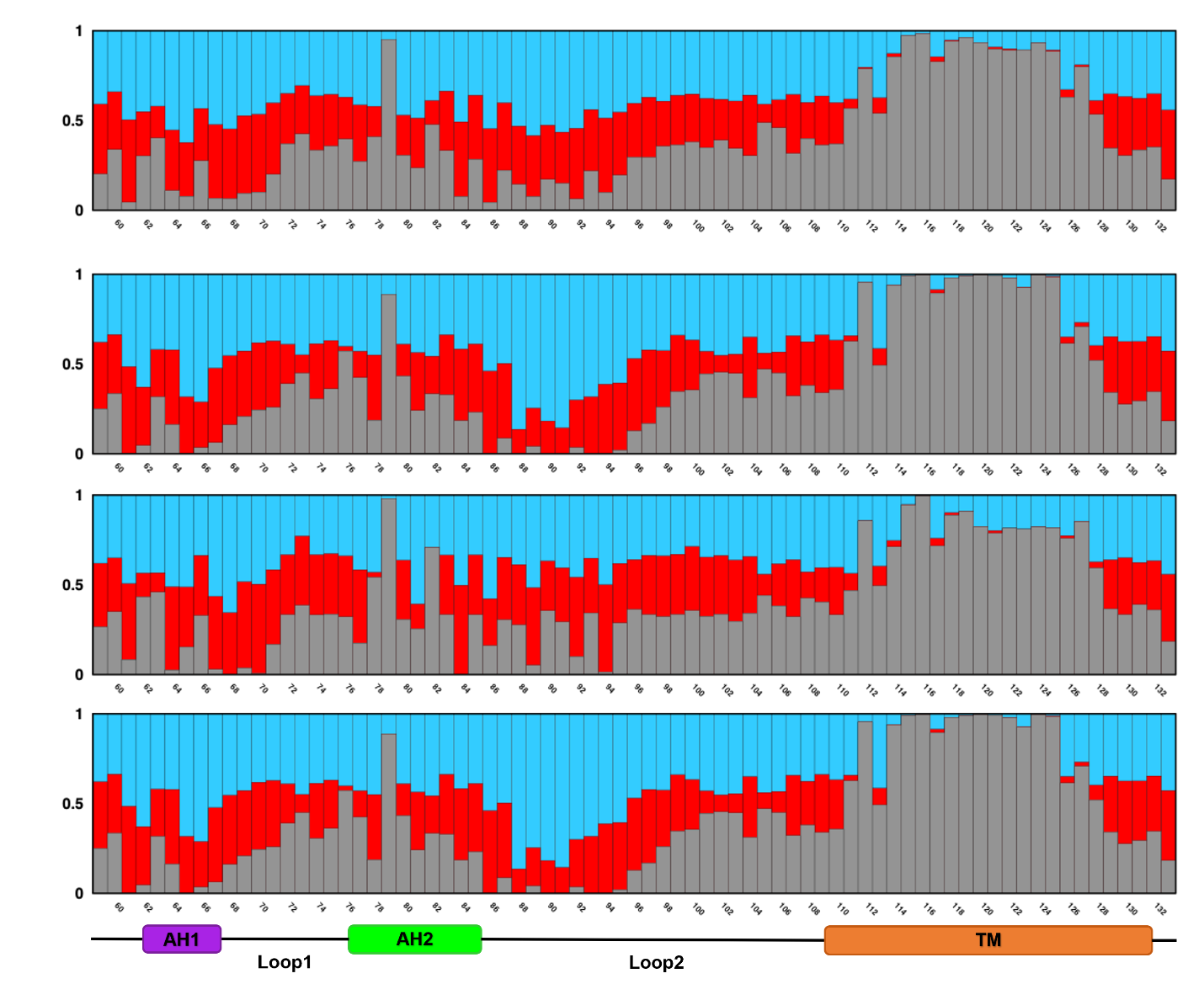
**Supplementary Figure 3. Interaction pattern of apo-IFITM3 in DMPC membrane system.** From the top to bottom: averaged among 3 replicas, replica #1, #2, and #3. Secondary structure of the protein is depicted at the bottom: purple for AH1, green for AH2 and orange for TM domain. Color codes: water (light blue), DMPC lipid head (red), and DMPC tail (gray).

**
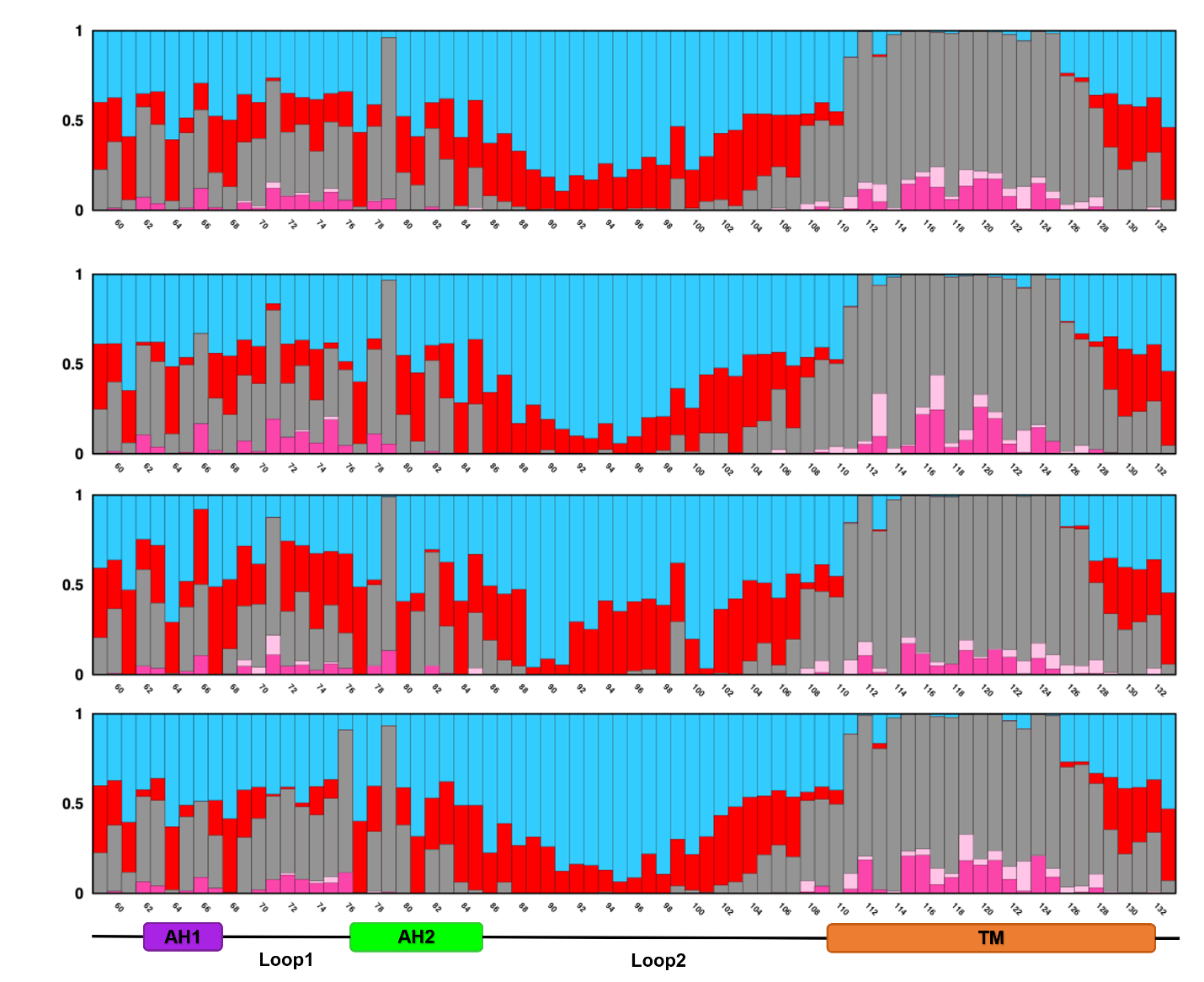
**

**Supplementary Figure 4. Interaction pattern of apo-IFITM3 in DMPC + Chol membrane system.** From the top to bottom: averaged among 3 replicas, replica #1, #2, and #3. Secondary structure of the protein is depicted on the bottom: purple for AH1, green for AH2 and orange for TM domain. Color codes: water (light blue), DMPC lipid head (red), or DMPC tail (gray), Chol headgroup (magenta), and Chol tail (light pink).

**
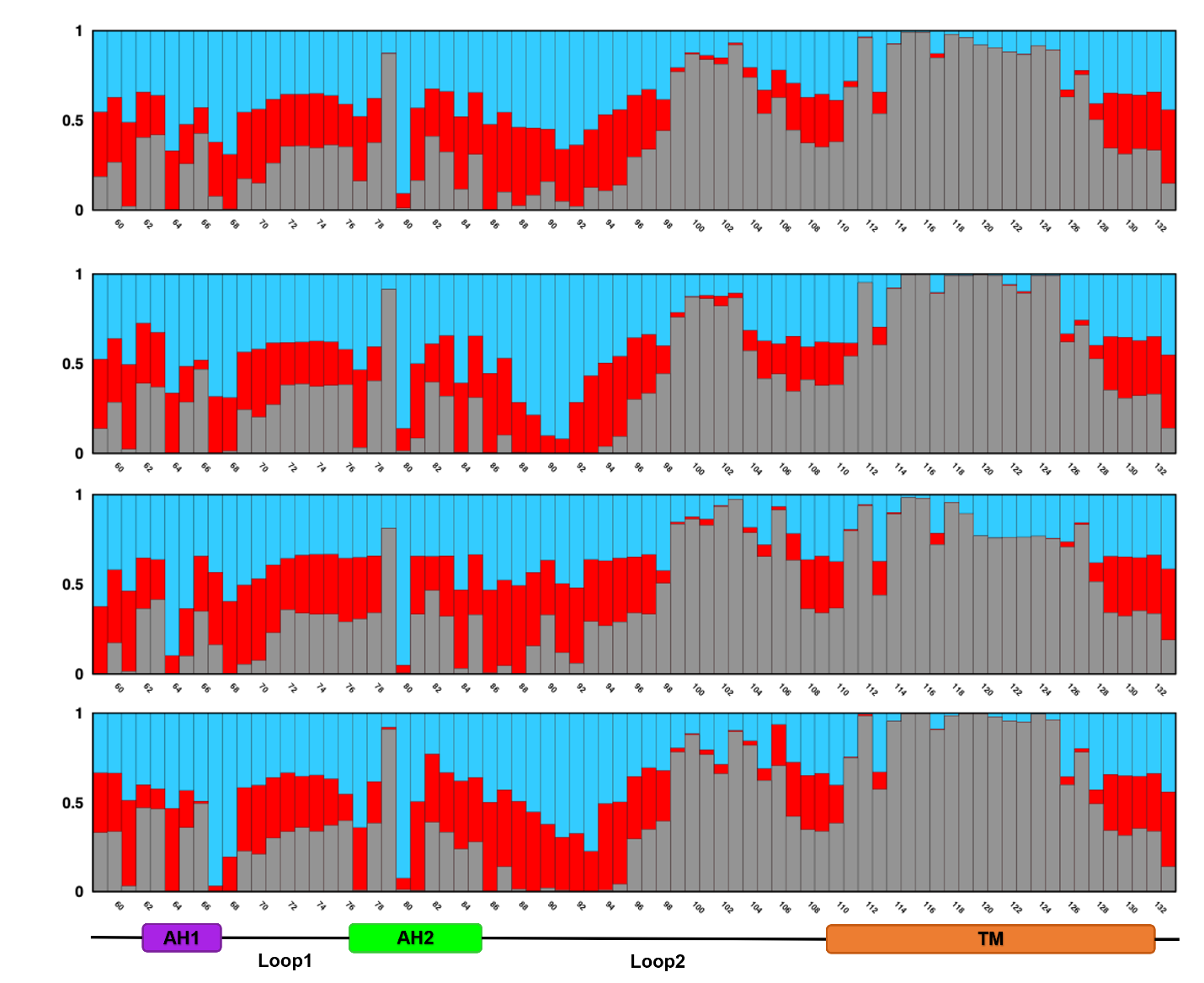
**

**Supplementary Figure 5. Interaction pattern of C72, 105 S-palmitoylated IFITM3 in DMPC membrane system.** From the top to bottom: averaged among 3 replicas, replica #1, #2, and #3. Secondary structure of the protein is depicted on the bottom: purple for AH1, green for AH2 and orange for TM domain. Color codes: water (light blue), DMPC lipid head (red), and DMPC tail (gray).

**
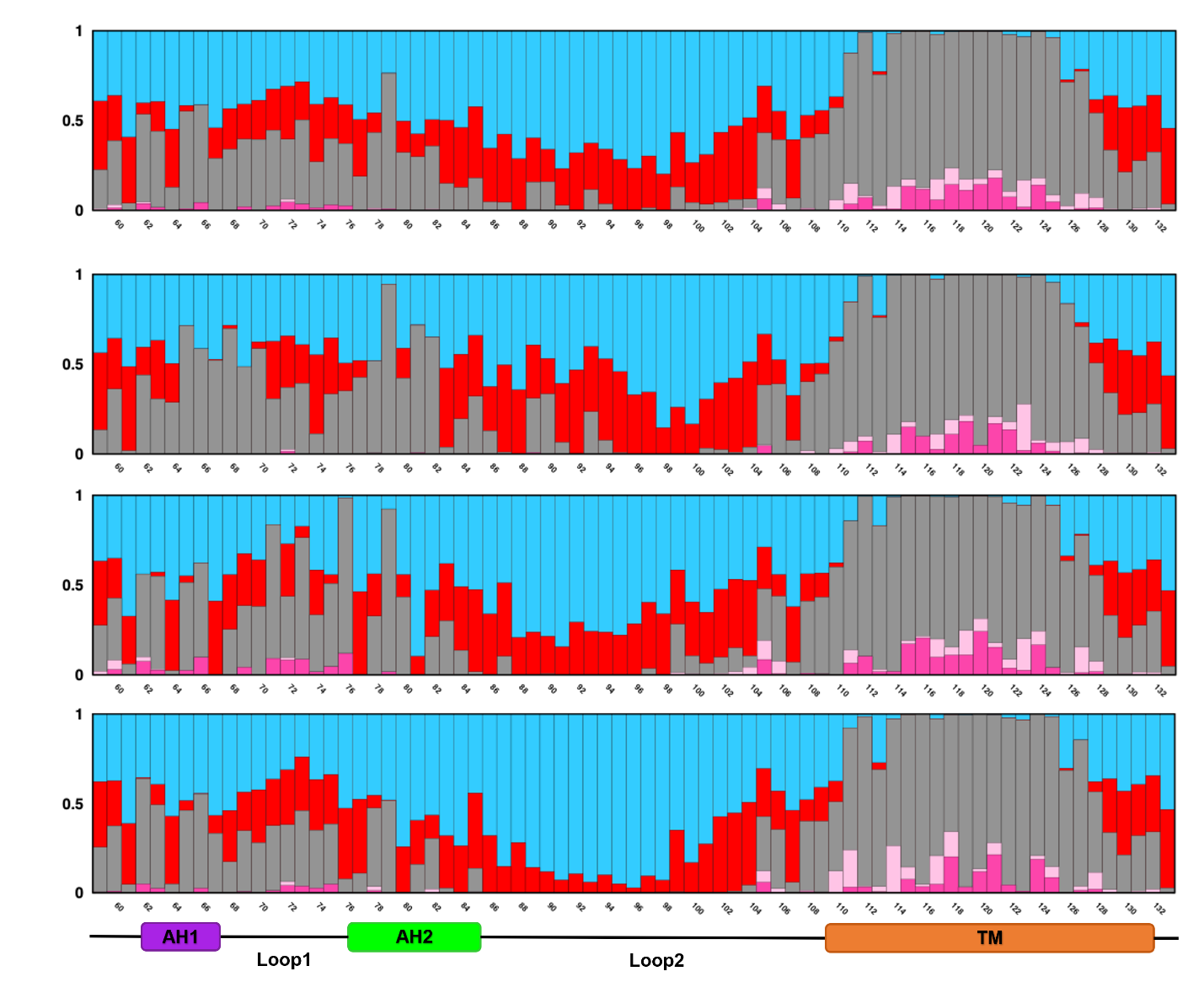
**

**Supplementary Figure 6. Interaction pattern of** **C72, 105 S-palmitoylated IFITM3 in DMPC + Chol membrane system.** From the top to bottom: averaged among 3 replicas, replica #1, #2, and #3. Secondary structure of the protein is depicted on the bottom: purple for AH1, green for AH2 and orange for TM domain. Color codes: water (light blue), DMPC lipid head (red), or DMPC tail (gray), Chol headgroup (magenta), and Chol tail (light pink).

**
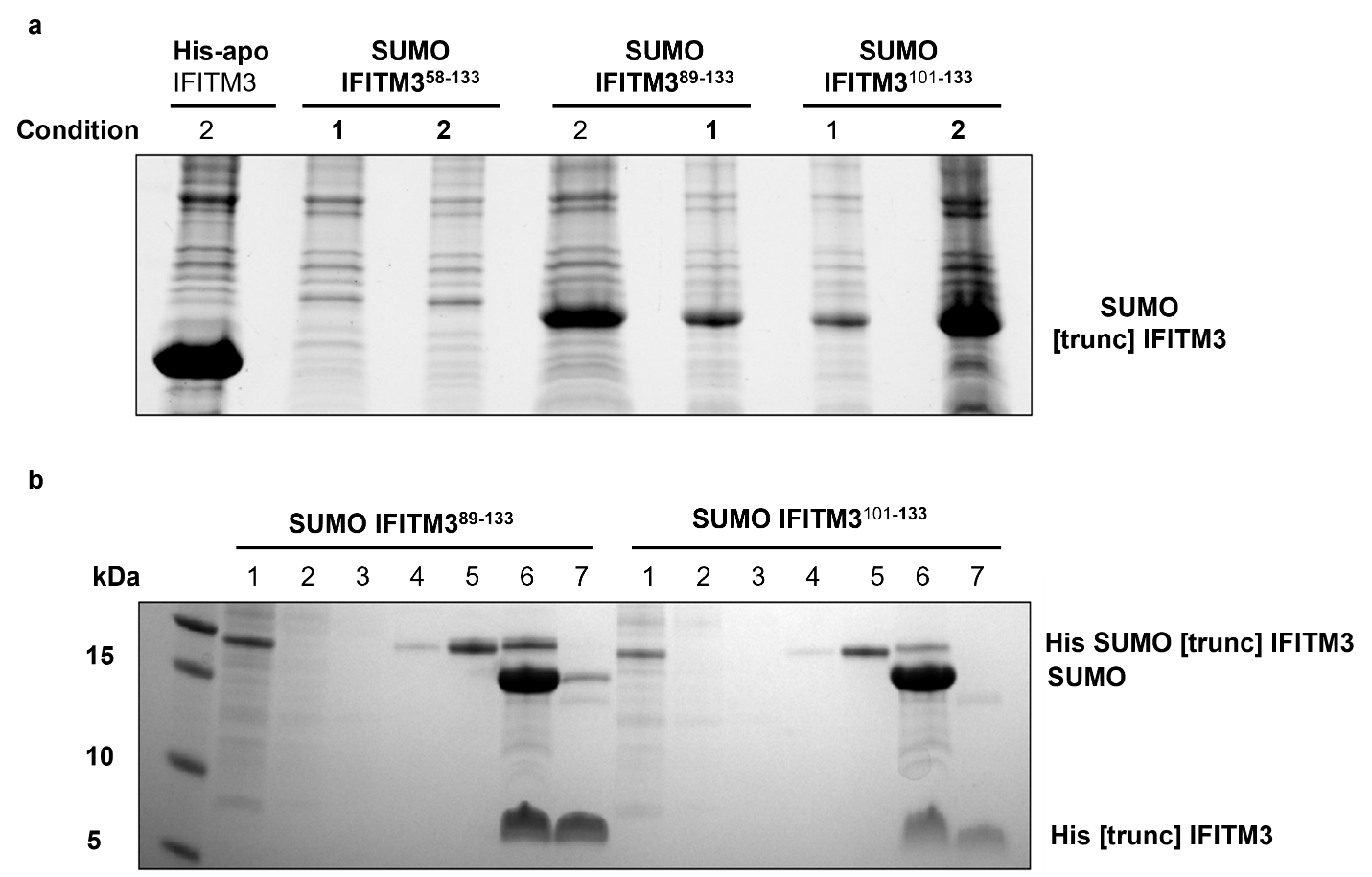
**

**Supplementary Figure 7. a,** Expression of the truncated IFITM3 construct in E. coli was tested by IPTG induction for 3 h at 37°C (condition 1) or overnight at 18°C (Condition 2). **b,** Purification of SUMO IFITM3^89-133^ and SUMO IFITM3^101-133^ from cell lysate by His-affinity purification. Lane key: “1” is the induced pellet, “2” is column flow through, “3” is wash 1, “4” is wash 2, “5” is column elution, “6” is ULP treated sample, “7” is sample with SUMO removed by incubation with cobalt beads.

**Supplementary Figure 8. Solution state NMR characterization of IFITM3 in membrane bicelle with cholesterol.** ^15^N-TROSY spectra of IFITM3^89-133^ in a DMPC (blue) and DMPC + 20% cholesterol (red) bicelles. Assigned peaks outside the hydrophobic core of the transmembrane domain are labeled. Structural perturbations found in the cholesterol rich environment are highlighted in boxes 1, 2, and 3.

**

**

**Supplementary Figure 9.** The synthetic route for bifunctional cholesterol reporter x-alk-chol.

**
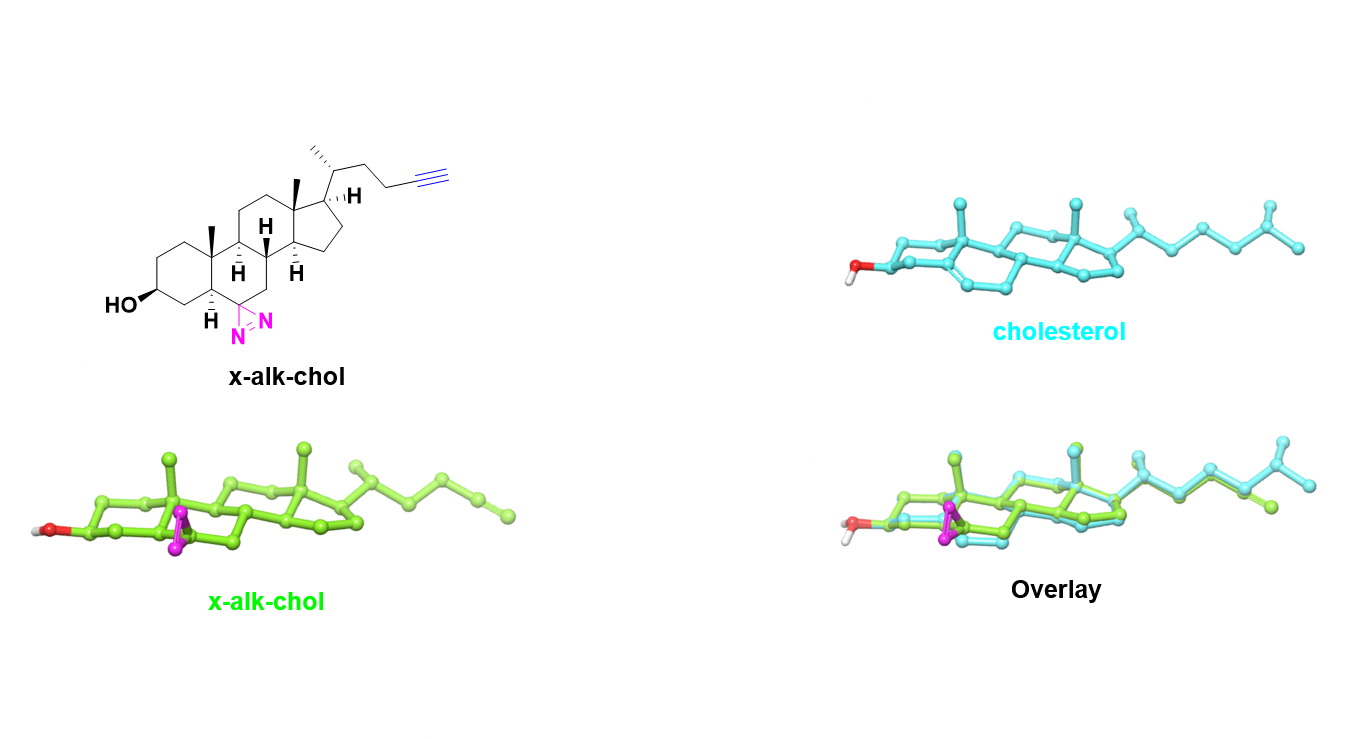
**

**Supplementary Figure 10. Maestro model of cholesterol and cholesterol probe x-alk-chol.** Maestro modeling shows topology of cholesterol and x-alk-chol.

**Supplementary Figure 11. In gel profiling of x-alk-chol labeled proteins in HeLa cell.** **a,** Experimental scheme for labeling of proteins with x-alk-chol in live cells. **b,** In-gel fluorescence profiling of x-alk-chol concentration dependent labeling of HeLa cell proteins. Cells were incubated with different concentrations of x-alk-chol for 30 min and irradiated with UV light (365 nm) for 5 min following which cell lysates were reacted with azide-rhodamine for fluorescence gel scanning. In-gel fluorescence profiling shows x-alk-chol concentration dependent labeling of proteins. α-Tubulin western blotting shows comparable protein loading. **c,** In-gel fluorescence profiling of cholesterol competition with x-alk-chol labeling of proteins. Cells were incubated with x-alk-chol for 30 min following which cells were treated with 10 μM cholesterol in mβCD and then irradiated with UV light (365 nm) for 5 min. Then cell lysates were reacted with azide-rhodamine for fluorescence gel scanning. In-gel fluorescence profiling shows cholesterol competition with x-alk-chol labeling of proteins. α-Actin western blotting shows comparable protein loading.


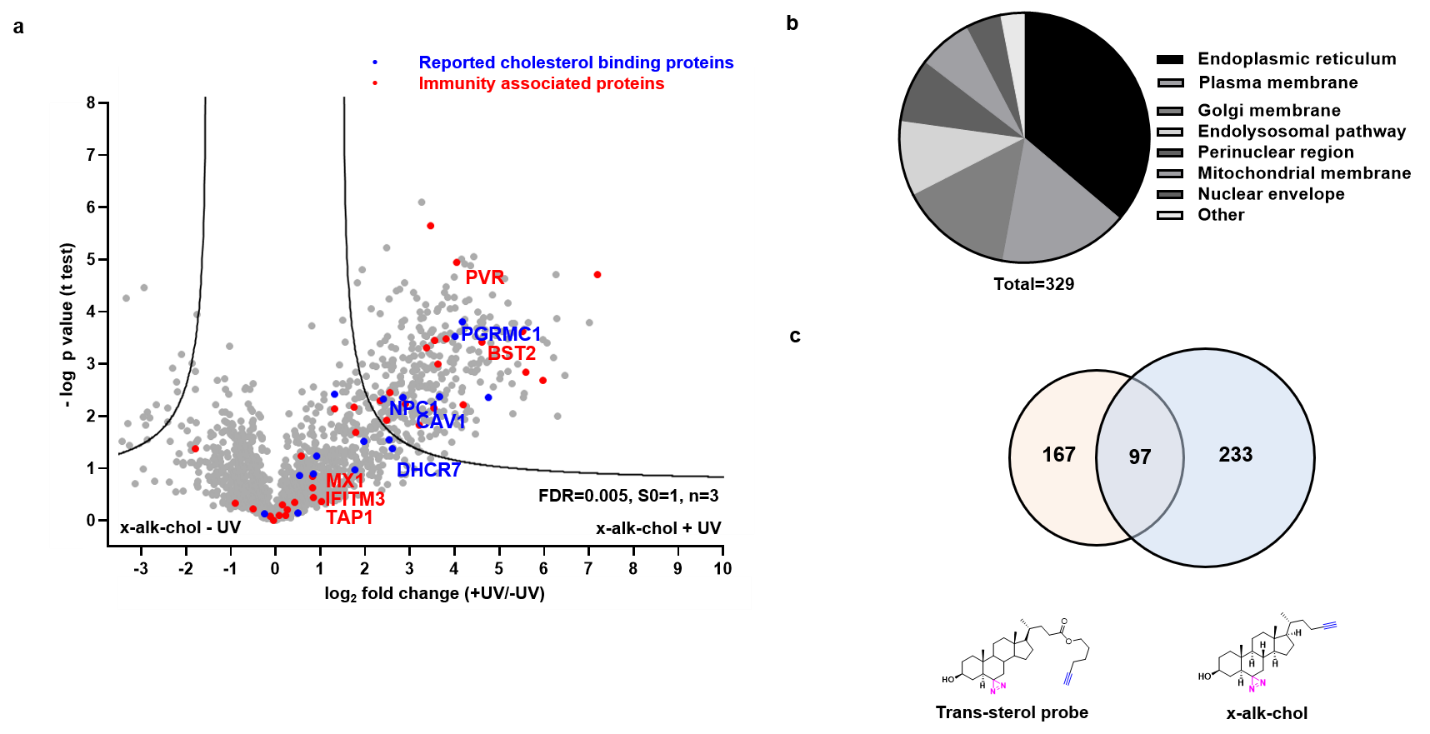


**Supplementary Figure 12. Proteomic analysis of x-alk-chol labeled proteins in Hela cell. a,** Volcano plot showing x-alk-chol binding proteins in HeLa cells. Cells were treated with x-alk-chol (10 μM) for 30 min and UV (365 nm) irradiation for 5 min. The cell lysates were further reacted with azide-biotin for enrichment of x-alk-chol labeled proteins with streptavidin beads and identification by mass spectrometry. Volcano plot shows enrichment of many reported cholesterol binding proteins (in blue) for UV treated sample. The x-axis is the difference of means between the UV treated and untreated samples and the y-axis is the log of the probability of that difference determined by the t-test. The minimum values for a valid protein are p<0.05 and a difference of means of 2 (FDR=0.0001, S0=1, n=3 replicates). **b,** Gene Ontology (GO) analysis shows subcellular localization of proteins enriched in UV treated samples. **c,** Venn Diagram shows overlap of protein hits from a previously published data set of Trans-sterol probe crosslinked proteins in Hela cells[^1^](https://sciwheel.com/work/citation?ids=1006176&pre=&suf=&sa=0&dbf=0).


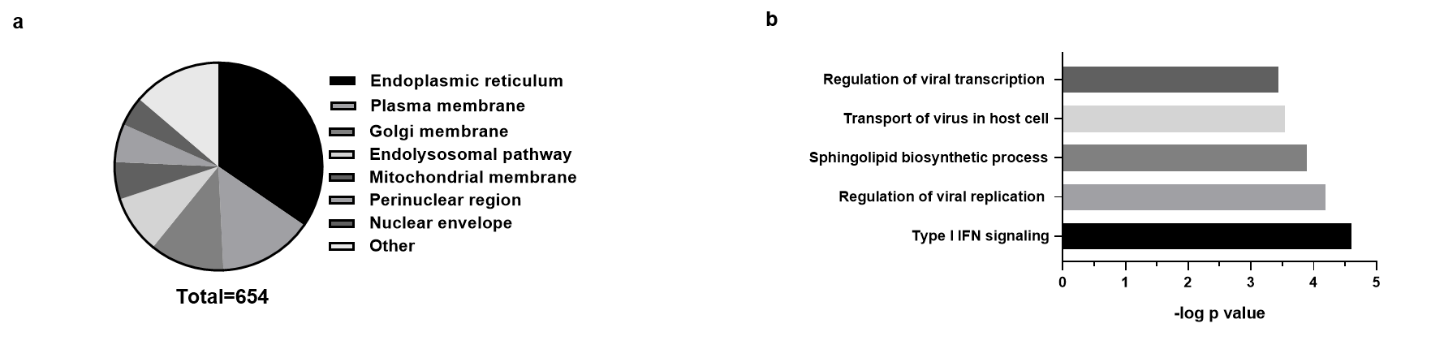


**Supplementary Figure 13. GO analysis of proteins enriched for UV treated IFN stimulated HeLa cell. a,** Gene Ontology (GO) analysis shows subcellular localization of proteins enriched in UV treated samples in Fig. 1c. **b,** GO analysis shows top 5 biological functions for proteins enriched only in UV treated IFN stimulated HeLa cells.


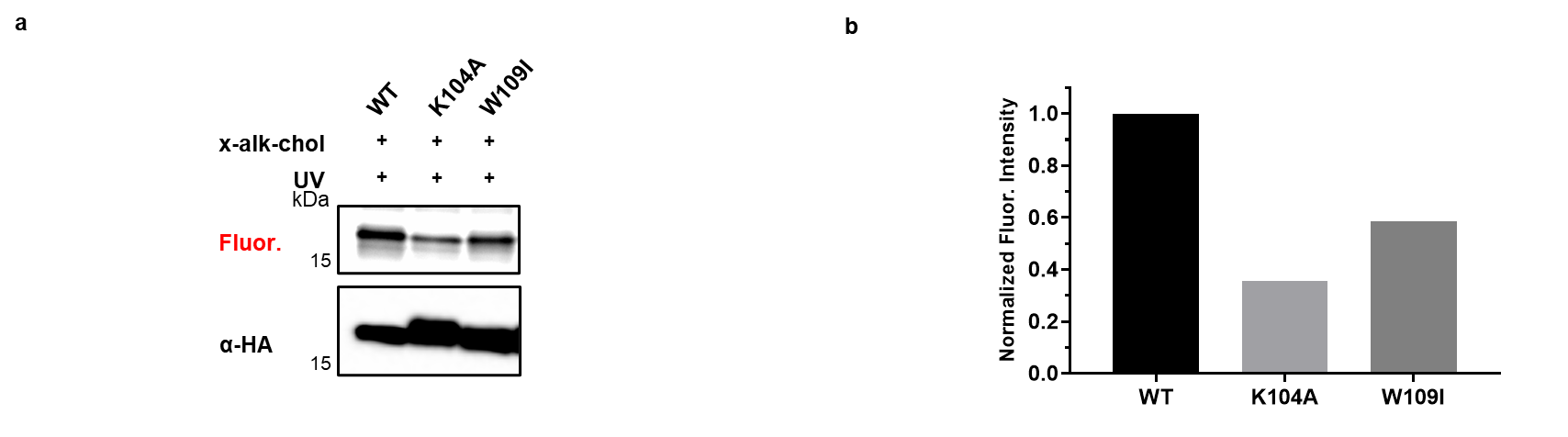


**Supplementary Figure 14. a, x-alk-chol labeling of overexpressed HA-tagged IFITM3 cholesterol binding mutants.** HEK293T cells were transfected with IFITM3 wildtype (WT), Lys104 to Ala and Trp109 to Ile constructs. Following 16 h transfection, cells were treated with x-alk-chol (10 µM) for 30 min and UV-irradiated for 5 min. The cell lysates were subjected to anti-HA immunoprecipitation and were further reacted with azide-rhodamine. In-gel fluorescence profiling shows lower fluorescence signal and therefore lower x-alk-chol labeling of IFITM3 Lys104 to Ala and Trp109 to Ile constructs. Anti-HA blot shows IFITM3 expression for each construct. **b,** Quantitative analysis of x-alk-chol labeling of the IFITM3 constructs in a. The fluorescence signal normalized to protein level was plotted for each construct.


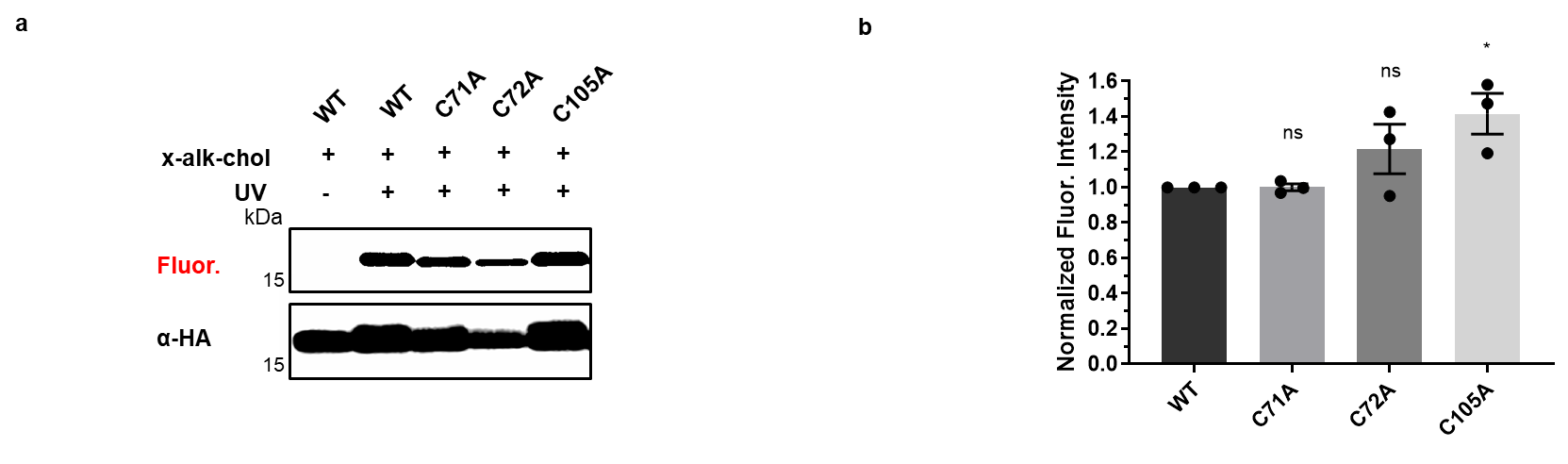


**Supplementary Figure 15. a, x-alk-chol labeling of overexpressed HA-tagged IFITM3 single Cys to Ala mutants.** HEK293T cells were transfected with IFITM3 wildtype (WT), single Cys71, 72, 105 to Ala constructs. Following 16 h transfection, cells were treated with x-alk-chol (10 µM) for 30 min and UV-irradiated for 5 min. The cell lysates were subjected to anti-HA immunoprecipitation and were further reacted with azide-rhodamine. In-gel fluorescence profiling shows no significant change in x-alk-chol labeling for Cys71, 72 to Ala mutants. IFITM3 Cys105 to Ala mutant shows slight increase in x-alk-chol labeling. Anti-HA blot shows IFITM3 expression for each construct. **b,** Quantitative analysis of x-alk-chol labeling of the IFITM3 constructs in a. The fluorescence signal normalized to protein level was plotted for each construct. Data represents the mean and s.e.m. of three independent experiments. P values were determined by one-way anova with a Dunnett's multiple comparisons test. *P<.05.


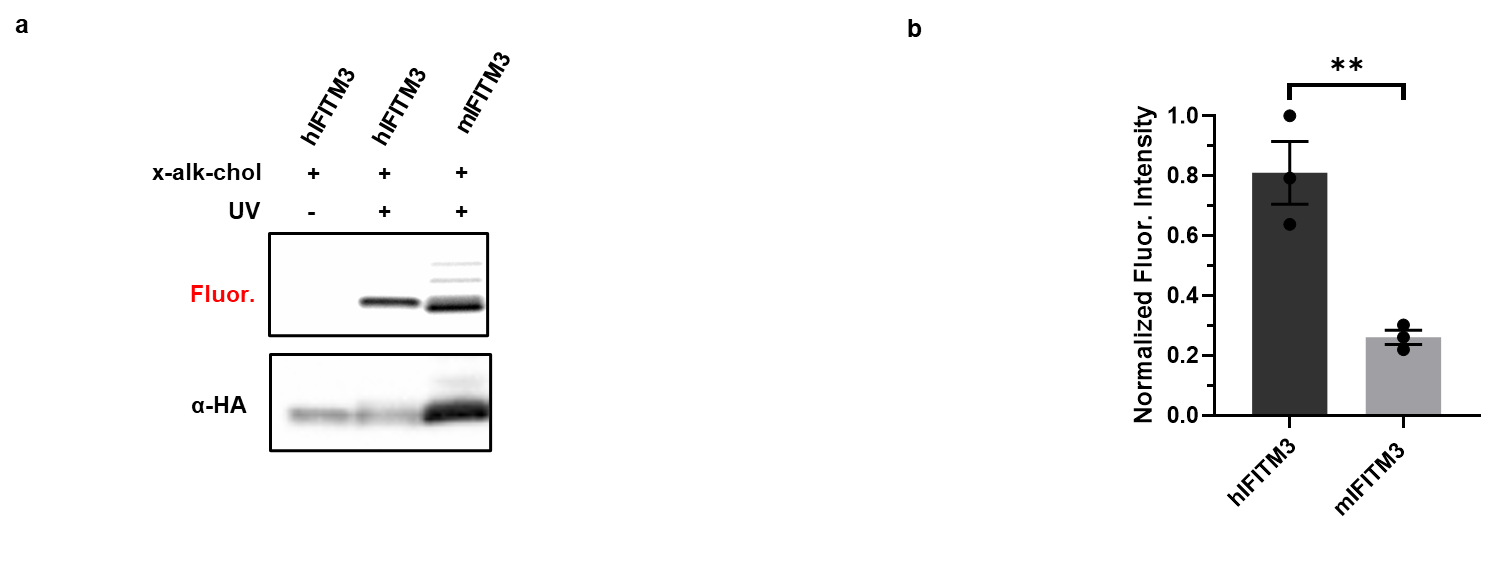


**Supplementary Figure 16. a,** x-alk-chol labeling of overexpressed human and mice IFITM3. HEK293T cells were transfected with HA-tagged human and mice IFITM3. Following 16 h transfection, cells were treated with x-alk-chol (10 µM) for 30 min and UV-irradiated for 5 min. The cell lysates were subjected to anti-HA immunoprecipitation and were further reacted with azide-rhodamine for in-gel fluorescence profiling. Anti-HA blot shows IFITM3 expression for each construct. **b,** Quantitative analysis of x-alk-chol labeling of the IFITM3 constructs in a. The fluorescence signal normalized to protein level was plotted for each construct. Data represents the mean and s.e.m. of three independent experiments. P values were determined by t-test. **P<.01.

**
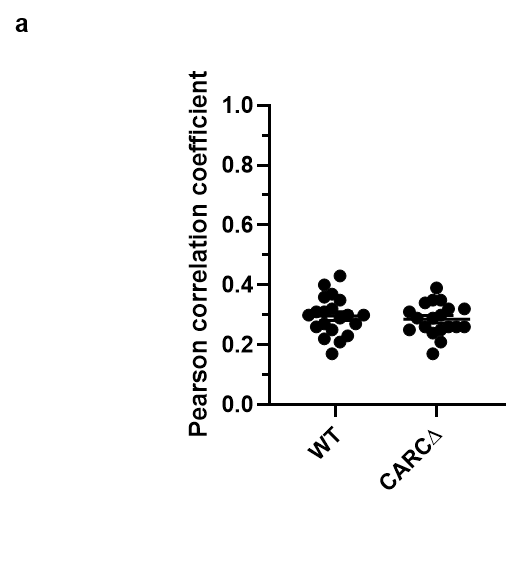
**

**Supplementary Figure 17. Quantification of myc-IFITM3 WT overlap with HA-IFITM3 WT or myc-IFITM3 CARCΔ in HeLa IFITM 2/3 KO cells.** Cells were fixed and stained with anti-HA antibody conjugated to Alexa Fluor 647 and anti-myc antibody conjugated to Alexa Fluor 488 and DAPI for nucleus. Quantification was performed by Image J. 20 cells were randomly picked from each group and the ratio of localization was analyzed under the same threshold. The percentage of merged volume above threshold colocalized was counted by Image J. The values shown represent the mean ± standard deviation (n = 20), as defined by error bars.

**
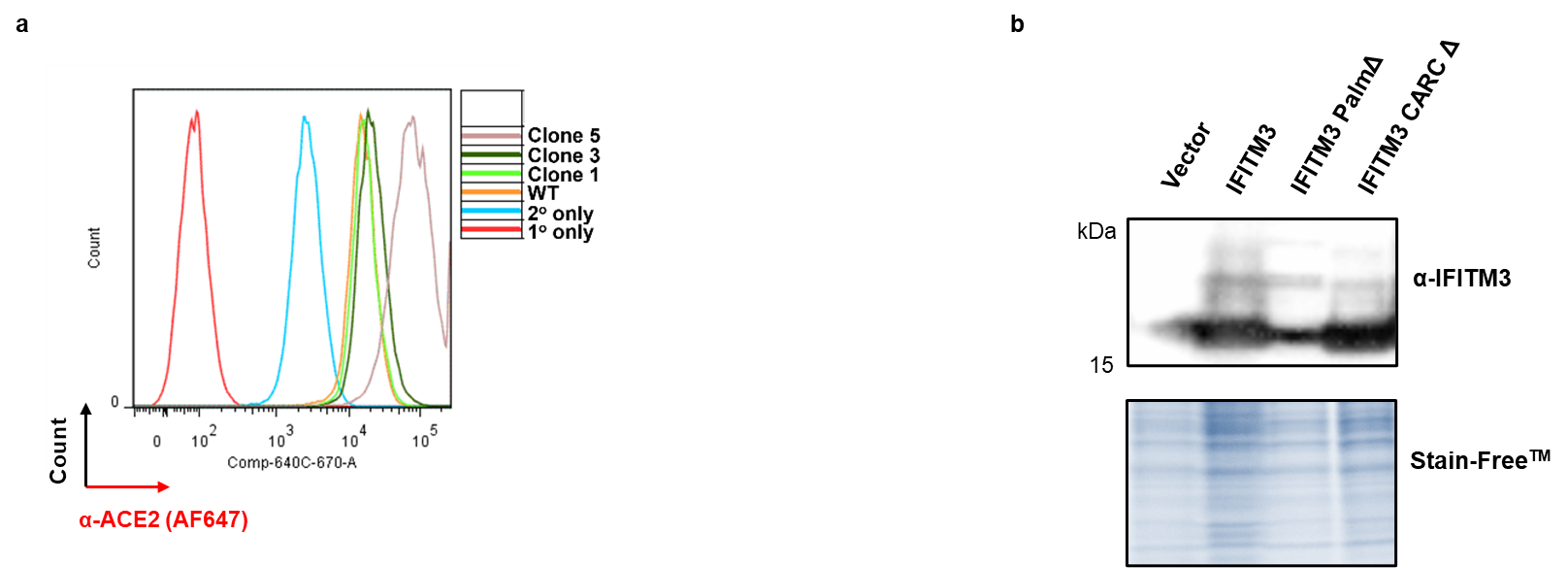
**

**Supplementary Figure 18. a,** Flow cytometry analysis of A549 IFITM1/2/3 KO-ACE2 cells for ACE2 expression level. **b,** Western Blot analysis of A549 IFITM1/2/3 KO-ACE2 cells expressing IFITM3 wildtype and mutants.


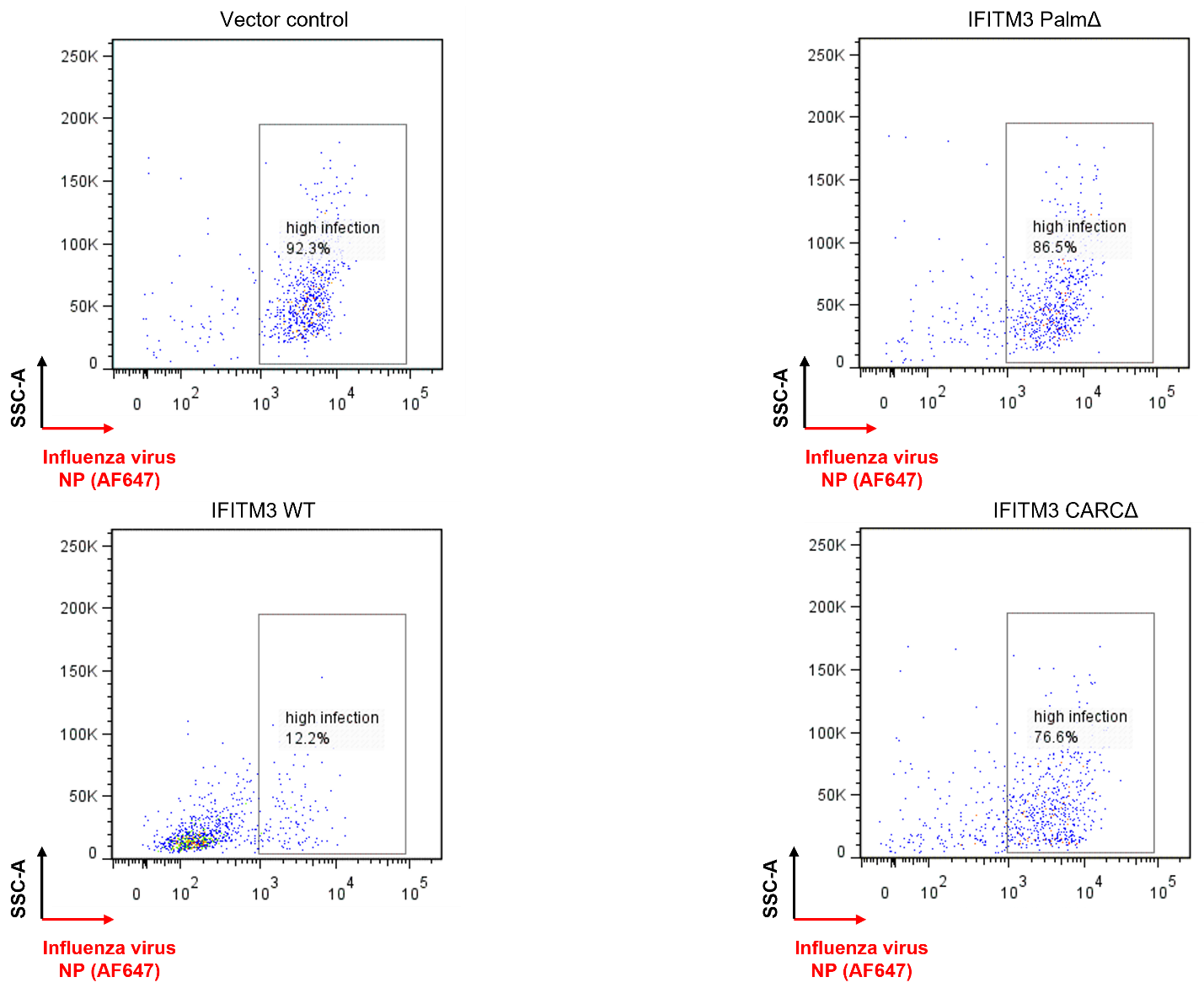


**Supplementary Figure 19. Representative flow cytometry data for IAV infection studies quantified in Fig. 2h.** Influenza A virus (IAV) infection of A549 IFITM1/2/3 KO cells stably expressing IFITM3 WT and cholesterol binding mutants. Cells were infected with IAV (MOI 10) for 6 h. Virus nucleoprotein (NP) levels were examined by flow cytometry using anti-NP staining and analyzed for percentage of infection.


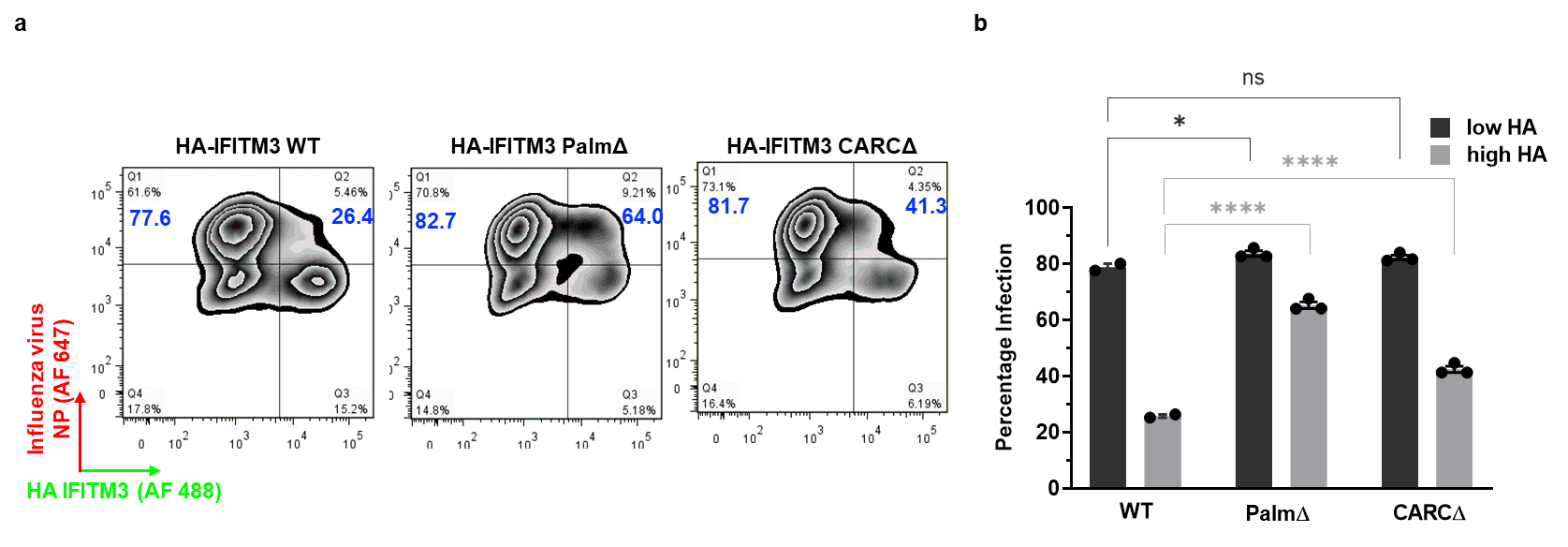


**Supplementary Figure 20.** **Representative flow cytometry data for IAV infection studies quantified in Fig. 2g.** **a,** Influenza A virus (IAV) infection of HEK293T cells expressing HA-tagged IFITM3 wildtype and cholesterol binding mutants. Cells were transfected with IFITM3 WT, PalmΔ and CARCΔ constructs following which cells were infected with IAV (MOI 2.5) for 6 h. Virus nucleoprotein (NP) and HA-IFITM3 protein levels were examined by flow cytometry using anti-NP and anti-HA staining, respectively. Non-transfected and transfected cells expressing IFITM3 from the same sample were gated on (low and high respectively) and analyzed for percentage of infection. **b,** Quantification of a, data represents mean and s.e.m. for three independent experiments. P values were determined by two-way anova with a Sidak's multiple comparisons test. ns 0.05<P, *P<.05, ****P<.0001.


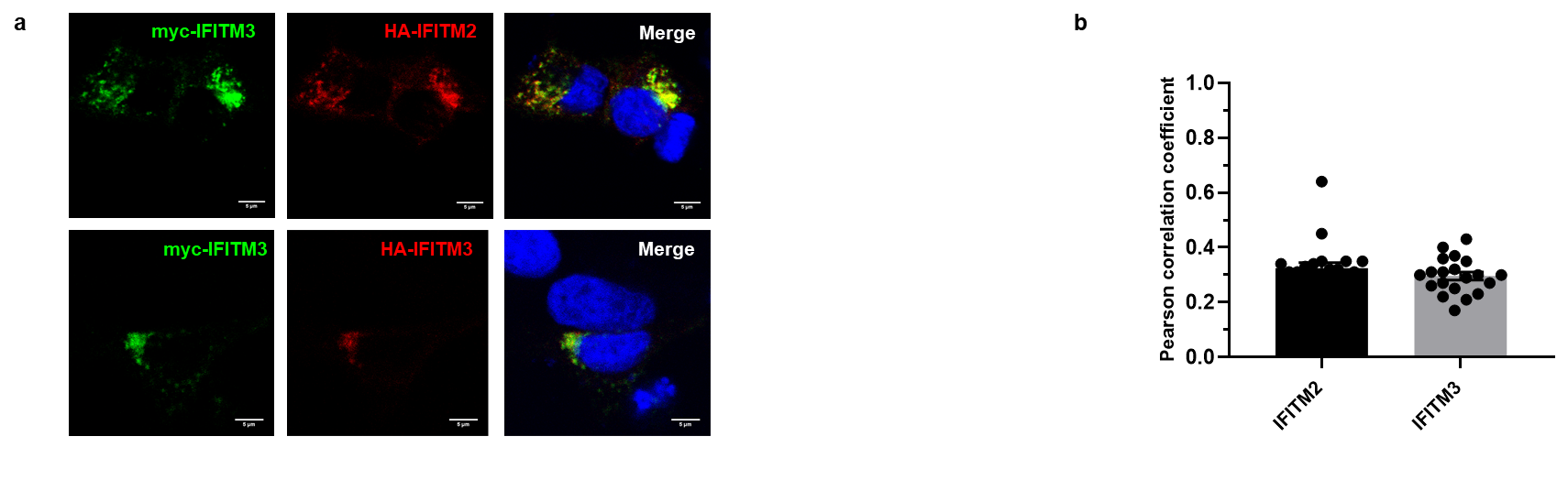


**Supplementary Figure 21. Quantification of myc-IFITM3 WT overlap with HA-IFITM isoforms and mutants in HeLa IFITM 2/3 KO cells. a,** Confocal microscopy of myc-IFITM3 (green) and HA-tagged IFITM co-transfected cells. HeLa IFITM 2/3 KO cells were transfected with myc-IFITM3 WT and HA- tagged IFITM constructs. Cells were fixed and stained with anti-HA antibody conjugated to Alexa Fluor 647 and anti-myc antibody conjugated to Alexa Fluor 488 and DAPI for nucleus. Column 1, localization of myc-IFITM3 WT (green) and HA-IFITM2 WT (red); Column 2, localization of myc-IFITM3 WT (green) and HA-IFITM3 WT (red); Column 3, localization of myc-IFITM3 WT (green) and HA-IFITM2-3C (red); Column 4, localization of myc-IFITM3 WT (green) and HA-IFITM3-2C (red). **b,** Quantification was performed by Image J. 20 cells were randomly picked from each group and the ratio of localization was analyzed under the same threshold. The percentage of merged volume above threshold colocalized was counted by Image J. The values shown represent the mean ± standard deviation (n = 20), as defined by error bars.


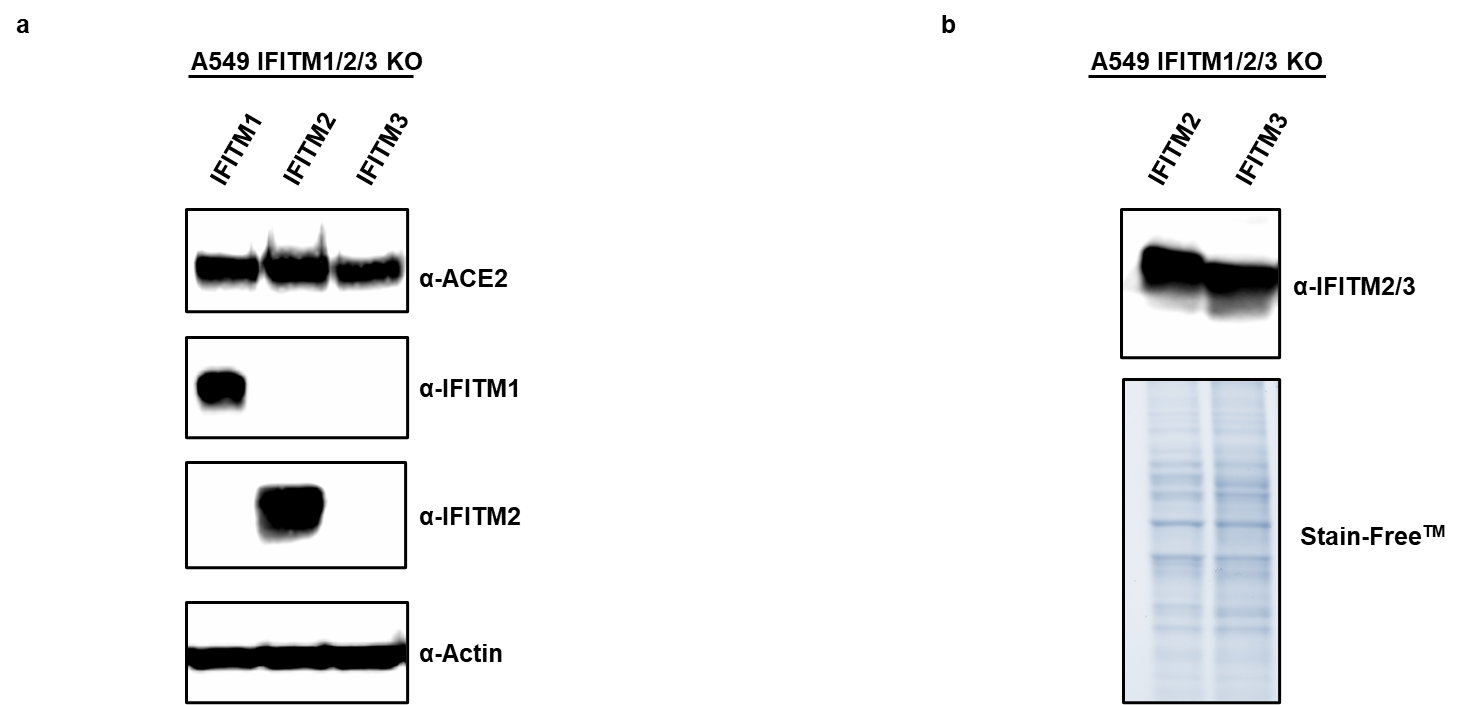


**Supplementary Figure 22. a, b,** Western Blot analysis of A549 IFITM1/2/3 KO-ACE2 cells expressing ACE2 and IFITM isoforms.

**Table S1. MD simulation system information.**

| **System** | **Lipid**  **Composition** | **Simulation**  **time** | **Repeats** |
| --- | --- | --- | --- |
| Apo-IFITM3 | DMPC | 2500 ns | 3 |
| C72, 105 *S*-Palmitoylated IFITM3 | DMPC | 1000 ns |  |
| Apo-IFITM3 | DMPC + Chol | 2000 ns |  |
| C72, 105 *S*-Palmitoylated IFITM3 | DMPC + Chol | 2000 ns |  |

**Table S2. Tilt angles comparison.**

|  |  | Apo-IFITM3 | | C72, 105 *S*-Palmitoylated IFITM3 | |
| --- | --- | --- | --- | --- | --- |
|  |  | DMPC | DMPC + Chol | DMPC | DMPC + Chol |
| **AH1** | **Tilt angles** | 101.53 | 86.85 | 108.4 | 77.7 |
|  | **(-) 90** | *+11.53* | *-3.15* | *+18.4* | *-12.3* |
| **AH2** | **Tilt angles** | 110.17 | 124.63 | 106.62 | 145.8 |
|  | **(-) 90** | *+20.17* | *+34.63* | *+16.62* | *+55.8* |

**Materials and Methods**

**Cell culture**

HeLa, HEK293T and A549 cells are from ATCC. Cells were cultured in 10% FBS (VWR) in DMEM (Gibco) at 37 °C with 5% CO_2_ and passaged every 2–3 days at 90% confluency.

**Viruses and Pseudo viruses**

Influenza A/PR/8/34 (H1N1) (10100374) was ordered from Charles River Laboratories. rVSV-eGFP SARS-CoV-2 S was generated following the protocol published previously[^2^](https://sciwheel.com/work/citation?ids=9409591&pre=&suf=&sa=0&dbf=0).

**Reagents**

IFITM1, 2, 3 antibodies were ordered from Cell Signaling Technology (13126S), Proteintech group (66137-1-Ig) and VWR International (11714-1-AP) respectively. Influenza A virus nucleoprotein antibody [AA5H] (ab20343) was from Abcam. AlexaFluor 647 Antibody Labeling Kit (A20186) was ordered from Life Technologies. Human interferon-α1 (8927SC) was purchased from Cell Signaling Technology. Anti-Caveolin-1 antibody was purchased from Cell Signaling Technology (3238S). Anti-HA HRP-conjugated antibody was ordered from Roche. The secondary antibodies HRP-conjugated goat anti-mouse IgG and HRP-conjugated goat anti-rabbit IgG (ab205718) were purchased from Cell Signaling Technology and Abcam respectively. HRP-conjugated Tubulin and Actin antibodies were purchased from Cell Signaling Technology. Anti-HA antibody conjugated to AlexaFluor-488, AlexaFluor-594 and AlexaFluor-647 were purchased from Life Technologies. Anti-Myc-Tag antibody conjugated to AlexaFluor-488 was purchased from Cell Signaling Technology (2279). Hoechst 33342, Trihydrochloride, Trihydrate (H3570) was purchased from Life Technologies. Isotopes (^15^N-ammonium sulfate, ^13^C6-glucose, D_2_O) were acquired from Cambridge Isotopes Laboratories, Inc. TALON cobalt resin was purchased from Takara Bio. Lipofectamine 3000 Transfection Reagent was purchased from Invitrogen. Trypsin was ordered from Promega. Streptavidin beads and Ezview anti-HA affinity gel were purchased from Thermo and Sigma Aldrich respectively. alk-16 and azido-rhodamine were synthesized in the lab following previously published protocol[^3^](https://sciwheel.com/work/citation?ids=791632&pre=&suf=&sa=0&dbf=0). Azido-biotin was purchased from Click Chemistry tools.

**Plasmids**

pCMV-HA-IFITM isoform constructs were used for transient transfection experiments. IFITM constructs were cloned into pLX304 for generation of A549 stable cells expressing IFITM isoforms. pLenti-hACE2-hygro was a gift from Neville Sanjana (Addgene plasmid # 161758). IFITM mutant plasmids were obtained with the QuikChange II Site-Directed Mutagenesis Kit (#200523) from Agilent Technologies. After site directed mutagenesis, all plasmids were DNA sequenced to verify their identity. IFITM3 was cloned into a pET28c-SUMO construct (gift from Lima lab, MSKCC). To develop truncations, Q5 amplification and ligation via KLD enzyme mix (NEB) was used to delete the desired protein domains.

**Metabolic labeling with x-alk-chol**

Hela cells were stimulated with IFN-α (100 µg/mL) or HEK293T cells transfected with HA-tagged IFITM constructs (1.5 μg/well) using 3 μl Lipofectamine 3000 in 1 mL of complete cell growth media. After 16 h, cells were incubated with 10 μM x-alk-chol in mβCD for 30 min. Cells were washed with PBS and UV treated for 5 min. Cells were lysed in 1% Brij 97 in 50 mM TEA, 150 mM NaCl pH 7.4, 1X Roche protease inhibitor, and 1,500 units/mL benzonase (EMD). Protein concentrations were determined by the BCA assay.

For immunoprecipitation studies with the cells expressing HA-tagged IFITMs, 150 μg of total protein was added to 20 μl of anti-HA antibody-conjugated agarose beads in a total volume of 150 μl and rocked at 4 °C for 1 h. Agarose beads were washed by resuspension in 500 μl of 50 mM HEPES buffer (containing 150 mM NaCl, pH 7.4) by centrifugation at 3,500 × *g* for 30 s. The beads were then resuspended in 45 μl of the above buffer and 5 μl of CuAAC reactant solution (0.5 μl of 10 mM azido-rhodamine (final concentration 100 μM), 1 μl of 50 mM freshly prepared CuSO4·5H2O in H2O (final concentration 1 mM), 1 μl of 50 mM freshly prepared TCEP (final concentration 1 mM) and 2.5 μl of 10 mM Tris[(1-benzyl-1H-1,2,3-triazol-4-yl)methyl]amine (TBTA) (final concentration 500 μM)) was added. The samples were rocked at room temperature for 1 h and washed twice with RIPA buffer. Laemmli sample buffer (20 μl) was added to the samples (1.0:1.3 ratio of buffer to sample), which were heated for 10 min at 95 °C and separated by gel electrophoresis. In-gel fluorescence scanning was performed using a Bio-Rad ChemiDoc MP Imaging System. Proteins separated by SDS-PAGE were transferred (50 mM Tris, 40 mM glycine, 0.0375% SDS, 20% MeOH in deionized water, Bio-Rad Trans-Blot Semi-Dry Cell, 25 V, 30 min) onto a nitrocellulose membrane which was blocked with 5% nonfat dried milk in PBS for 1 h at 25 °C. The membrane was incubated with anti-HA HRP conjugated antibody (diluted 1:1,000 in PBST) overnight at 4 °C, subsequently washed three times with PBST (0.1% Tween-20 in PBS), and developed with ECL Western blotting detection reagents (Biorad). Quantification of band intensities in fluorescence gels and western blots were performed with Image Lab (Bio-Rad). Data from three biological replicates were quantified and averaged for plotting using GraphPad Prism.

**Proteomic analysis**

HeLa cells were grown to confluency in 10 cm dishes. Cells were stimulated with IFN-α (100 µg/mL) overnight. Next cells were treated with x-alk-chol (10 μM) for 30 min and UV treated for 5 min. Cells were lysed with 4% SDS in 50 mM HEPES buffer with 150 mM NaCl. 2 mg of cell lysate was reacted with azido-biotin for 4 h (1 mM CuSO_4_, 1 mM TCEP, 200 μM TBTA, 200 μM azido-biotin) at 1 mg/mL. Protein was precipitated using methanol (8 mL), chloroform (3 mL), and water (6 mL) overnight at −20 °C. Protein pellets were dried for 1 h and solubilized in 200 μL of 4% SDS buffer. The sample was diluted to 1 mg/mL with 50 mM HEPES buffer. 25 μL of streptavidin beads were added to the samples and were nutated for 1 h. The beads were washed with 1% SDS, 5 M Urea, and PBS. Samples were split and half the beads were boiled in 20 μL of 4% SDS loading buffer and separated by SDS-PAGE for western blot analysis. The other half was suspended in 200 μL of 25 mM ammonium bicarbonate, reduced with 1 mM DTT for 30 min, and then alkylated with 50 mM iodoacetamide in the dark for 30 min. Then, the beads were washed with 200 μL of 25 mM ammonium bicarbonate and suspended in 50 μL of 25 mM ammonium bicarbonate. 0.1 μg of trypsin was added, and the samples were digested at 37 °C overnight. The supernatant was collected and dried on a speedvac and solubilized in 5% acetonitrile/1% formic acid for LC-MS/MS analysis.

Extracted tryptic peptides were desalted on a trap column following separation on a 12 cm/75 μm reversed phase C_18_ column (Nikkyo Technos Co., Ltd. Japan). A 180-minute gradient increasing from 10% B to% 45% B in 133 minutes (A: 0.1% Formic Acid, B: Acetonitrile/0.1% Formic Acid) were delivered at 200 nL/min. The liquid chromatography setup (Dionex, Boston, MA, USA) was connected to an Orbitrap XL (Thermo, San Jose, CA, USA) operated in top-8-CID-mode with MS spectra measured at a resolution of 60,000@m/z 400. Acquired tandem MS spectra were extracted using Maxquant queried against the Uniprot complete human database and processed using Perseus. Absent values were imputed based on the normal distribution of values. To determine if a protein was a valid hit, the false discovery rate was lower than 0.01% and the mean difference of the control and labeled samples had be greater than 2 with p<0.05 determined by the t-test. Database for Annotation, Visualization and Integrated Discovery (DAVID) was used for Gene Ontology (GO) enrichment analysis.

**Metabolic labeling with alkyne-palmitic acid reporter alk-16**

HEK293T cells were transfected with HA-tagged IFITM constructs (1.5 μg/well) using 3 μl Lipofectamine 3000 in 1 mL of complete cell growth media. After 16 h, cells were incubated with 50 μM alk-16 in DMEM containing 10% FBS for 2 h. Cells were harvested, washed once with PBS and lysed in 1% Brij 97 (Sigma) in 50 mM TEA, 150 mM NaCl pH 7.4, 1X Roche protease inhibitor, and 1,500 units/mL benzonase (EMD). Protein concentrations were determined by the BCA assay. For immunoprecipitation, 150 μg of total protein was added to 20 μL of anti-HA antibody-conjugated agarose in a total volume of 150 μl and rocked at 4 °C for 1 h. Agarose beads were washed by resuspension in 500 μl of 50 mM HEPES buffer (containing 150 mM NaCl, pH 7.4) by centrifugation at 3,500 × *g* for 30 s. The beads were then resuspended in 45 μl of the above buffer and 5 μl of CuAAC reactant solution (0.5 μl of 10 mM), azido-rhodamine (final concentration 100 μM), 1 μl of 50 mM freshly prepared CuSO4·5H2O in H2O (final concentration 1 mM), 1 μl of 50 mM freshly prepared TCEP (final concentration 1 mM) and 2.5 μl of 10 mM Tris[(1-benzyl-1H-1,2,3-triazol-4-yl)methyl]amine (TBTA) (final concentration 500 μM)) was added. The samples were rocked at room temperature for 1 h and washed twice with RIPA buffer. Laemmli sample buffer (20 μl) was added to the samples (1.0:1.3 ratio of buffer to sample), which were heated for 10 min at 95 °C and separated by gel electrophoresis. In-gel fluorescence scanning was performed using a Bio-Rad ChemiDoc MP Imaging System. Western blots for HA-tagged proteins were performed using an anti-HA HRP conjugated antibody (1:1,000; Roche). Quantification of band intensities in fluorescence gels and western blots were performed with Image Lab (Bio-Rad). Data from three biological replicates were quantified and averaged for plotting using GraphPad Prism.

**Stable cell protocol**

HEK293T cells were seeded in 10 cm dish. They were transfected with plasmid encoding protein of interest and lentiviral packaging and envelope-expressing plasmid. After 6h, media was changed. After 40 h, supernatant was collected and centrifuged at 1200g for 5 min. After filtering the supernatant through a 0.45 μM membrane, it was aliquoted and frozen at -80 ^o^C.

A549 IFITM1/2/3 KO cells were treated with the supernatant diluted 1:1 in polybrene containing media. After two days, cells were treated with the appropriate antibiotic to select for transduced cells. A549 IFITM 1/2/3 KO- ACE2 cells were further sorted to get single clones.

**Influenza A virus infection**

HEK293T cells were seeded in 12 well plates and cultured overnight. Cells were co-transfected with HA-tagged IFITM constructs (1.5 μg/well) using 3 μL Lipofectamine 3000 in 1 mL of complete cell growth media. After 16 h, cells were infected with influenza virus A/PR/8/34 (H1N1). After 6 h of infection, cells were trypsinized and collected in cluster tubes. Cells were washed twice with PBS and then fixed with 400 μl of 4% PFA in PBS for 10 min. The fixed cells were permeabilized with 200 μl of 0.2% saponin in PBS for 10 min and then blocked with 200 μl 0.2% BSA and 0.2% saponin in PBS for 10 min. Cells were treated with anti-influenza NP antibody conjugated to AlexaFluor-647 and anti-HA antibody conjugated to Alexafluor-488 (1:250). After three washes with PBS, cells were resuspended in 150 μl of 0.2% BSA and 0.02% saponin in PBS. The samples were analyzed by flow cytometry (BD LSRII). All samples were first gated by HA-positive staining, indicating successful transfection, and then NP-positive staining, indicating successful infection. The HA-tag epitope is derived from an H3 influenza virus strain and is not present in H1N1 influenza virus. Data analysis was performed using FlowJo software.

**Pseudo virus infection**

A549 cells were plated on 96-well plates (Corning) 18-24 h prior to infection. Cells were infected with pre-titrated amounts of rVSV-eGFP SARS-CoV-2 S from viral supernatant that was optimized for 20-40% infection, as described previously[^2^](https://sciwheel.com/work/citation?ids=9409591&pre=&suf=&sa=0&dbf=0). At 7 h post-infection, the cells were fixed with 4% paraformaldehyde (Alfa Aesar), washed with PBS, and stained with 1:5000 dilution of PBS containing Hoechst-33342 (Invitrogen). Viral infectivity was measured using a Cytation5 automated fluorescent microscope (BioTek) to count the eGFP-positive cells to estimate % infected cells by using the Gen5 data analysis software (BioTek).

**Confocal microscopy**

HeLa cells were seeded on precision cover glasses (thickness No. 1.5H) in 6-well plates. After 24 h, cells were transfected with HA-tagged and Myc-tagged IFITM constructs for 16 h. Cells were then fixed with 4% formaldehyde in PBS for 10 min, permeabilized with 0.5% saponin in PBS for 10 min, blocked with 1% BSA in PBS for 1 h and immunostained with anti-HA (1:100) and anti-Myc (1:100) antibodies conjugated to Alexa Fluor 488 and Alexa Fluor 647, respectively. Finally, the coverslips were mounted onto slides using ProLong™ Diamond Antifade Mountant containing DAPI. The cells were imaged on an inverted LSM 780 laser scanning confocal microscope (Zeiss) with a Zeiss Plan-Apochromatic 63×/1.4 oil immersion objective. Images were taken under identical confocal microscope settings. DAPI was excited with a 405 nm laser. Alexa Fluor 488 was excited with a 488 nm laser and Alexa Fluor 647 was excited with a 633 nm laser. Images were acquired with the ZEN blue 2012 software (Zeiss) and analyzed by ImageJ (NIH). Pearson's correlation coefficients were calculated in ImageJ using the Coloc 2 plugin.

**Molecular dynamics simulation set-up**

Based on our previous study[^4^](https://sciwheel.com/work/citation?ids=10025411&pre=&suf=&sa=0&dbf=0), initial position and each isoform structure of IFITM3 were prepared. For system building, the CHARMM36[^5–7^](https://sciwheel.com/work/citation?ids=321550,920807,11519170&pre=&pre=&pre=&suf=&suf=&suf=&sa=0,0,0&dbf=0&dbf=0&dbf=0) force field for protein and lipids was used for the potential energy calculations and CHARMM-GUI[^8,9^](https://sciwheel.com/work/citation?ids=763245,4406911&pre=&pre=&suf=&suf=&sa=0,0&dbf=0&dbf=0) *Membrane Builder*[^10–13^](https://sciwheel.com/work/citation?ids=1887706,1127587,7909816,1643016&pre=&pre=&pre=&pre=&suf=&suf=&suf=&suf=&sa=0,0,0,0&dbf=0&dbf=0&dbf=0&dbf=0) was utilized for the whole system building process. 41 CHOL molecules were added to CHOL-containing system with 164 DMPC (1,2-dimyristoyl-*sn*-glycero-3-phosphocholine) molecules on upper leaflet, and 39 CHOL molecules were added to the lower leaflet with 156 DMPC molecules. The final system dimension is ~110 x 110 x 90 Å^3^. The DMPC pure bilayer membrane contains 94 DMPC lipids on both upper and lower leaflets with the system dimension of ~80 x 80 x 100 Å^3^. TIP3P water model[^14^](https://sciwheel.com/work/citation?ids=324142&pre=&suf=&sa=0&dbf=0)and 150mM KCl ions were added to mimic the ionic conditions of cellular environment. Equilibration processes were conducted based on the *Membrane Builder*’s standard protocol, and OpenMM 7.5 package[^15^](https://sciwheel.com/work/citation?ids=5474011&pre=&suf=&sa=0&dbf=0) was used for the production simulation (see the simulation time in **Supplementary Table 1**). The van der Waals interactions were controlled by a force-based switching method[^16^](https://sciwheel.com/work/citation?ids=11519661&pre=&suf=&sa=0&dbf=0)with 10 to 12 angstrom cut-off range. For long-range electrostatic interactions, the particle-mesh Ewald method[^17^](https://sciwheel.com/work/citation?ids=925696&pre=&suf=&sa=0&dbf=0) was employed, and the SHAKE algorithm[^18^](https://sciwheel.com/work/citation?ids=453035&pre=&suf=&sa=0&dbf=0) was used to fix bonds including hydrogen atom. The temperature was held at 308.15 K for all systems by Langevin dynamics[^19^](https://sciwheel.com/work/citation?ids=11519478&pre=&suf=&sa=0&dbf=0) with 1 bar pressure that was maintained under the semi-isotropic Monte-Carlo barostat method[^20^](https://sciwheel.com/work/citation?ids=11519401&pre=&suf=&sa=0&dbf=0)[^21^](https://sciwheel.com/work/citation?ids=9326979&pre=&suf=&sa=0&dbf=0) with a 5 ps^-1^ coupling frequency. To improve the conformational sampling, 3 replica simulations were conducted for each system and the last 500 nano-second trajectories were used for all analyses.

**IFITM3 expression and reconstitution into bicelles**

To express and purify isotopically labeled truncated IFITM3, a fusion SUMO-IFITM3 construct was designed to prevent proteolytic cleavage in *E. coli*. In brief, *E. coli* BL21 cells transformed with a pET28a-His-SUMO-IFITM3^trunc^ plasmid were inoculated in a 3 ml culture which was spun down and resuspended in 50ml M9 minimal media (MM) after 12 hours. For ^2^H^13^C^15^N-labeled samples, this culture was adjusted to 100% D_2_O M9 MM over the course of a day. At this point, the culture was inoculated into 1L of isotopically labeled MM (either ^15^N-ammonium chloride for ^15^N-labeled samples, or ^15^N-ammonium chloride, ^13^C-glucose, and D_2_O for triple labeled samples used for backbone assignment). Cells were grown at 37°C and 220 rpm until the culture reached an OD_600_ between 0.8 and 0.9. At this point the culture was induced with 1mM IPTG and grown overnight at 18°C.

After expression, the cells were lysed in 25 mM HEPES, 150 mM KCl, and 2% TX-100 after which the lysate was clarified by ultracentrifugation. The lysate was diluted to a final TX-100 concentration of 0.8% and incubated with TALON cobalt resin for immobilized metal affinity purification (IMAC). The protein was eluted in 25 mM HEPES, 150mM KCl, 400 mM imidazole, and 1% octyl-*β*-glucopyranoside after which it was concentrated and incubated with ULP1 for SUMO domain cleavage. After dialysis to remove imidazole, the protein was co-incubated with TALON cobalt resin a second time to remove the His-tagged SUMO domain from the final protein prep.

To incorporate truncated IFITM3 into bicelles, lipid was prepared by dissolving in 20 mM sodium phosphate and 20 mM NaCl at 15 mg/mL. A homogenous solution was achieved through approximately 20 freeze-thaw cycles, or until the solution was uniformly cloudy with no particulates, after which 100 μL of 1 M DTT was added to each mL of lipid. Lipid was mixed with protein in 10 mL 1% OG containing buffer at a ratio of 1:50 protein to lipid, after which the protein and lipid solution was incubated at room temperature for 1 hour with gentle shaking. 2.5 g biobeads were added to solution and incubated for 1 h at room temperature, followed by incubation overnight at 4 ^o^C. In the morning, 1 g biobeads were added for 1 h to remove any remaining detergent before the liposome solution was ultracentrifuged at 100,000 xg for 1.5 h to pellet. The lipid pellet was resuspended in 25 mM HEPES and 150 mM KCl, pH 7 followed by approximately five freeze-thaw cycles to buffer exchange. The liposomes were then ultracentrifuged at 150,000xg for 2.5 h followed by resuspension in DHPC buffer. The q value was measured using ^1^H NMR and adjusted to 0.5-0.6.

The NMR data was collected on Bruker 800 and 900 MHz AVANCE spectrometers equipped with TCI CryoProbes at 37°C. ^15^N- traverse relaxation optimized spectroscopy (TROSY) spectra were collected on ^15^N-labeled IFITM3^89-133^ in DMPC and DMPC + cholesterol bicelles to observe changes in the protein backbone chemical shift. In order to assign the soluble regions of the protein, triple resonance TROSY experiments (^15^N-HSQC, HNCA, HNCO, HNCACB, HNCOCA, HNCOCACB, and HNCACO) were performed on ^2^H^13^C^15^N-labeled IFITM3^89-133^ in deuterated DMPC bicelles.

**Synthesis of x-alk-chol**

The precursor of the probe was synthesized according to previously published protocols[^1^](https://sciwheel.com/work/citation?ids=1006176&pre=&suf=&sa=0&dbf=0). The ketone precursor (50 mg) was dissolved in 7 N NH_3_ in MeOH (10 mL) at 0 ^o^C. The reaction mixture was stirred at 0 ^o^C for 3 h. Then hydroxylamine-O-sulfonic acid (1.5 eq) was added at -78 ^o^C. The resulting solution was stirred to room temperature overnight. The reaction mixture was filtered and washed with MeOH. The resulting filtrate was concentrated under reduced pressure and re-dissolved in MeOH/DCM (3:1, 10 mL). I_2_ (1.0 eq) and NEt_3_ (2 eq) were added at 0 ^o^C and stirred for 3 h. The reaction mixture was concentrated and washed with DCM/ sat. aq Na_2_S_2_O_3_. The organic extracts were dried over Na_2_SO_4_ and concentrated under reduced pressure. The residue was purified by CombiFlash chromatography to provide the desired probe x-alk-chol (28.9 mg, 56%). ^1^H-NMR (600 MHz, CDCl_3_, representative signals) δ (ppm) 3.46-3.44 (m, 1H), 2.24-2.20 (m, 1H), 2.12-2.09 (m, 1H), 2.08-2.06 (m, 1H), 1.1 (s, 3H), 0.92 (d, 3H, *J* = 4.5 Hz), 0.70 (s, 3H), 0.47-0.43 (m, 2H); ^13^C-NMR (150 MHz, CDCl_3_) 85.20, 71.20, 68.09, 56.14, 56.03, 53.82, 45.40, 42.93, 39.84, 37.68, 37.46, 36.46, 35.21, 34.87, 33.98, 33.29, 30.83, 29.47, 28.13, 24.02, 21.34, 18.24, 15.62, 13.11, 12.20.

**
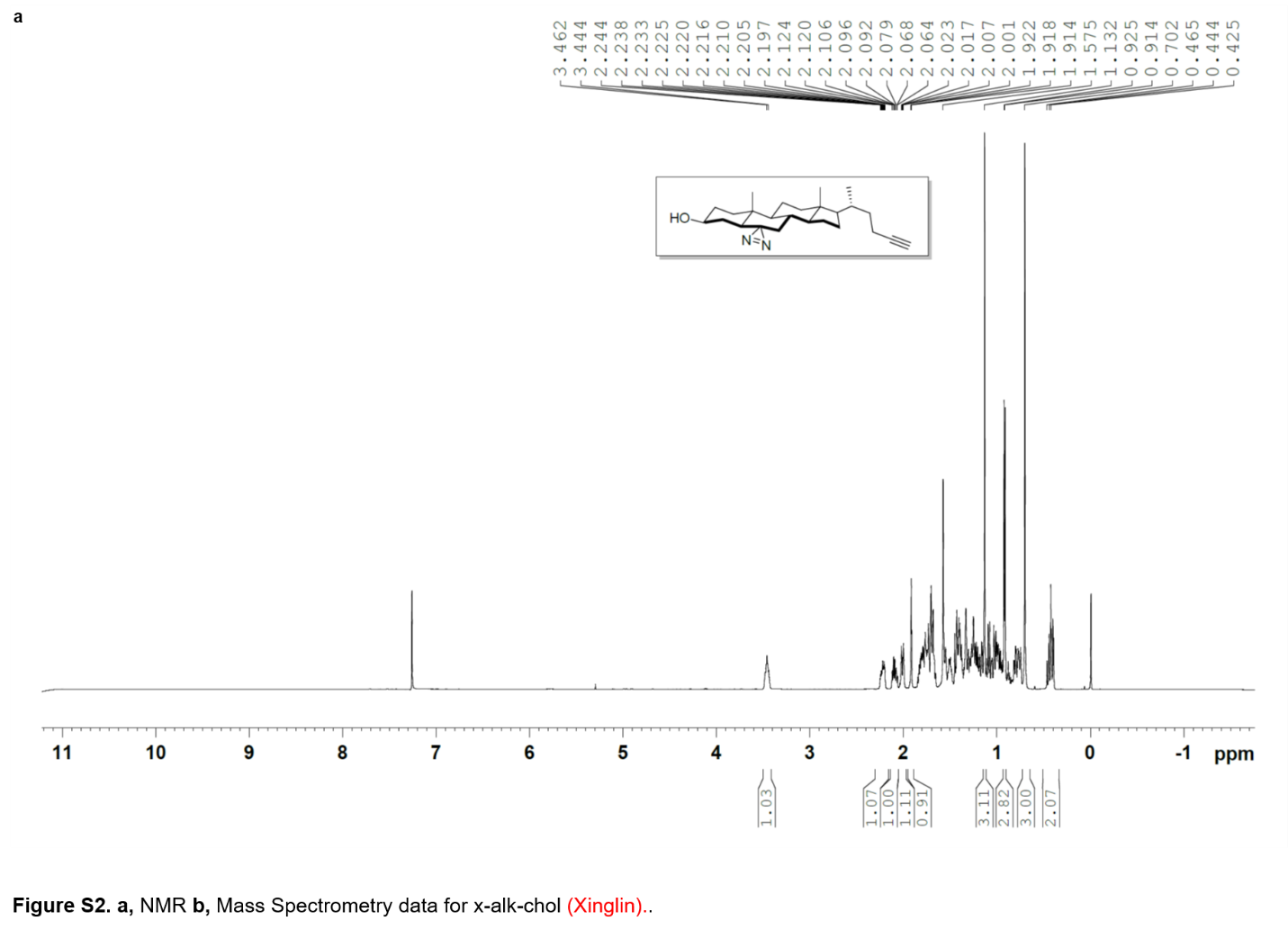
**

**
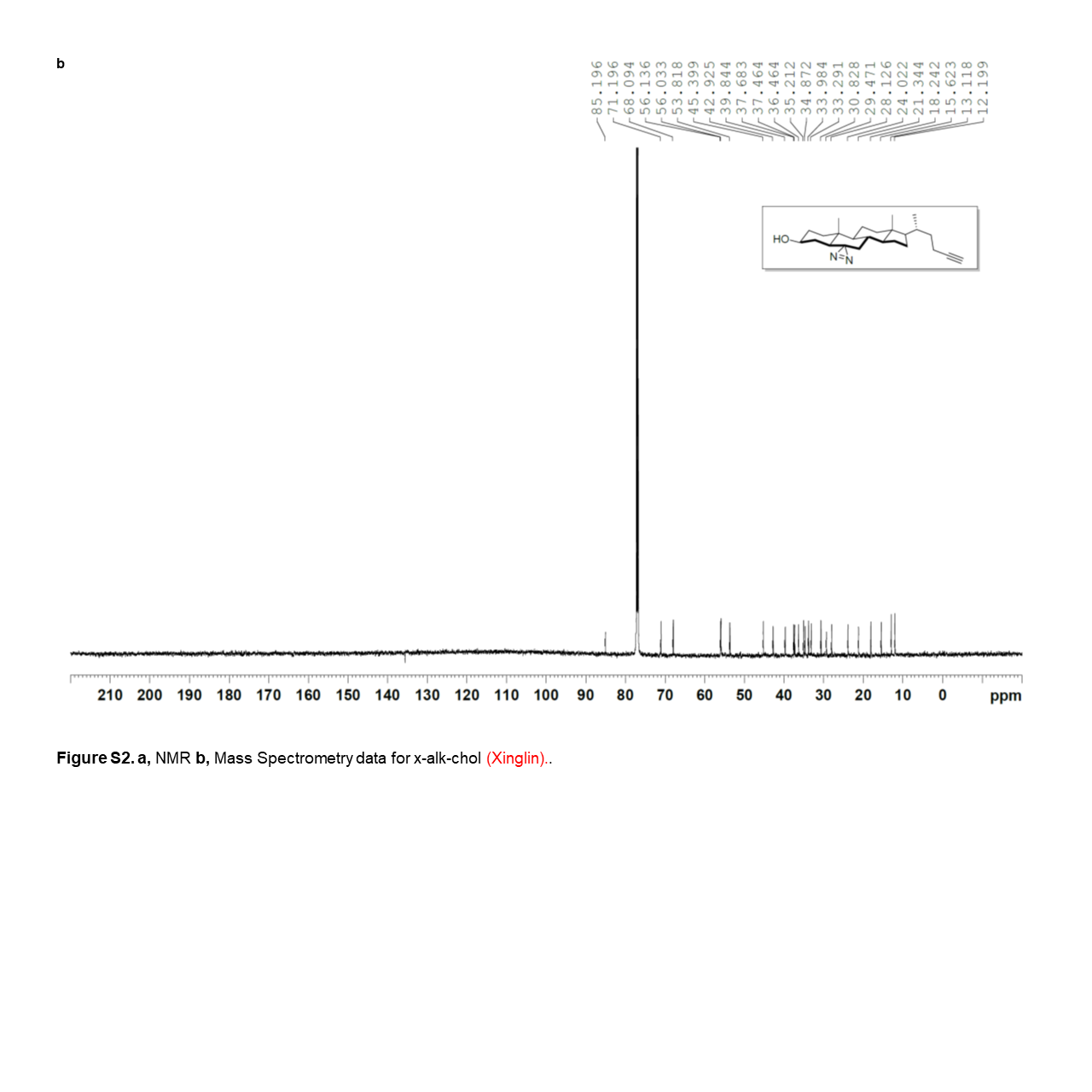
**

**Supplementary Figure 23. NMR spectra of x-alk-chol.** a) 1H-NMR spectra of x-alk-chol in CDCl_3_. b) 13C-NMR spectra of x-alk-chol in CDCl_3_.

**References**

[1.    Hulce, J. J., Cognetta, A. B., Niphakis, M. J., Tully, S. E. & Cravatt, B. F. Proteome-wide mapping of cholesterol-interacting proteins in mammalian cells. *Nat. Methods* **10**, 259–264 (2013).](https://sciwheel.com/work/bibliography/1006176)

[2.    Dieterle, M. E. *et al.* A Replication-Competent Vesicular Stomatitis Virus for Studies of SARS-CoV-2 Spike-Mediated Cell Entry and Its Inhibition. *Cell Host Microbe* **28**, 486-496.e6 (2020).](https://sciwheel.com/work/bibliography/9409591)

[3.    Yount, J. S. *et al.* Palmitoylome profiling reveals S-palmitoylation-dependent antiviral activity of IFITM3. *Nat. Chem. Biol.* **6**, 610–614 (2010).](https://sciwheel.com/work/bibliography/791632)

[4.    Garst, E. *et al.* Site-specific lipidation enhances IFITM3 membrane interactions and antiviral activity. *BioRxiv* (2020) doi:10.1101/2020.09.11.293324.](https://sciwheel.com/work/bibliography/10025411)

[5.    Brooks, B. R. *et al.* CHARMM: the biomolecular simulation program. *J. Comput. Chem.* **30**, 1545–1614 (2009).](https://sciwheel.com/work/bibliography/321550)

[6.    Klauda, J. B. *et al.* Update of the CHARMM all-atom additive force field for lipids: validation on six lipid types. *J. Phys. Chem. B* **114**, 7830–7843 (2010).](https://sciwheel.com/work/bibliography/920807)

[7.    Venable, R. M. *et al.* CHARMM all-atom additive force field for sphingomyelin: elucidation of hydrogen bonding and of positive curvature. *Biophys. J.* **107**, 134–145 (2014).](https://sciwheel.com/work/bibliography/11519170)

[8.    Jo, S., Kim, T., Iyer, V. G. & Im, W. CHARMM-GUI: a web-based graphical user interface for CHARMM. *J. Comput. Chem.* **29**, 1859–1865 (2008).](https://sciwheel.com/work/bibliography/763245)

[9.    Jo, S., Kim, T. & Im, W. Automated builder and database of protein/membrane complexes for molecular dynamics simulations. *PLoS ONE* **2**, e880 (2007).](https://sciwheel.com/work/bibliography/4406911)

[10.   Jo, S., Lim, J. B., Klauda, J. B. & Im, W. CHARMM-GUI Membrane Builder for mixed bilayers and its application to yeast membranes. *Biophys. J.* **97**, 50–58 (2009).](https://sciwheel.com/work/bibliography/1887706)

[11.   Wu, E. L. *et al.* CHARMM-GUI Membrane Builder toward realistic biological membrane simulations. *J. Comput. Chem.* **35**, 1997–2004 (2014).](https://sciwheel.com/work/bibliography/1127587)

[12.   Lee, J. *et al.* CHARMM-GUI Membrane Builder for Complex Biological Membrane Simulations with Glycolipids and Lipoglycans. *J. Chem. Theory Comput.* **15**, 775–786 (2019).](https://sciwheel.com/work/bibliography/7909816)

[13.   Lee, J. *et al.* CHARMM-GUI Input Generator for NAMD, GROMACS, AMBER, OpenMM, and CHARMM/OpenMM Simulations Using the CHARMM36 Additive Force Field. *J. Chem. Theory Comput.* **12**, 405–413 (2016).](https://sciwheel.com/work/bibliography/1643016)

[14.   Jorgensen, W. L., Chandrasekhar, J., Madura, J. D., Impey, R. W. & Klein, M. L. Comparison of simple potential functions for simulating liquid water. *J. Chem. Phys.* **79**, 926 (1983).](https://sciwheel.com/work/bibliography/324142)

[15.   Eastman, P. *et al.* OpenMM 7: Rapid development of high performance algorithms for molecular dynamics. *PLoS Comput. Biol.* **13**, e1005659 (2017).](https://sciwheel.com/work/bibliography/5474011)

[16.   Steinbach, P. J. & Brooks, B. R. New spherical-cutoff methods for long-range forces in macromolecular simulation. *J. Comput. Chem.* **15**, 667–683 (1994).](https://sciwheel.com/work/bibliography/11519661)

[17.   Essmann, U. *et al.* A smooth particle mesh Ewald method. *J. Chem. Phys.* **103**, 8577 (1995).](https://sciwheel.com/work/bibliography/925696)

[18.   Ryckaert, J.-P., Ciccotti, G. & Berendsen, H. J. C. Numerical integration of the cartesian equations of motion of a system with constraints: molecular dynamics of n-alkanes. *J. Comput. Phys.* **23**, 327–341 (1977).](https://sciwheel.com/work/bibliography/453035)

[19.   Goga, N., Rzepiela, A. J., de Vries, A. H., Marrink, S. J. & Berendsen, H. J. C. Efficient algorithms for langevin and DPD dynamics. *J. Chem. Theory Comput.* **8**, 3637–3649 (2012).](https://sciwheel.com/work/bibliography/11519478)

[20.   Chow, K.-H. & Ferguson, D. M. Isothermal-isobaric molecular dynamics simulations with Monte Carlo volume sampling. *Comput. Phys. Commun.* **91**, 283–289 (1995).](https://sciwheel.com/work/bibliography/11519401)

[21.   Åqvist, J., Wennerström, P., Nervall, M., Bjelic, S. & Brandsdal, B. O. Molecular dynamics simulations of water and biomolecules with a Monte Carlo constant pressure algorithm. *Chem. Phys. Lett.* **384**, 288–294 (2004).](https://sciwheel.com/work/bibliography/9326979)
