# Supplementary figures and images for: S-palmitoylation and sterol interactions mediate antiviral specificity of IFITM isoforms
Source: Res Sq. 2021 Dec 29:rs.3.rs-1179000. Preprint. [Version 1] doi: 10.21203/rs.3.rs-1179000/v1 (PMC8722608; doi:10.21203/rs.3.rs-1179000/v1)

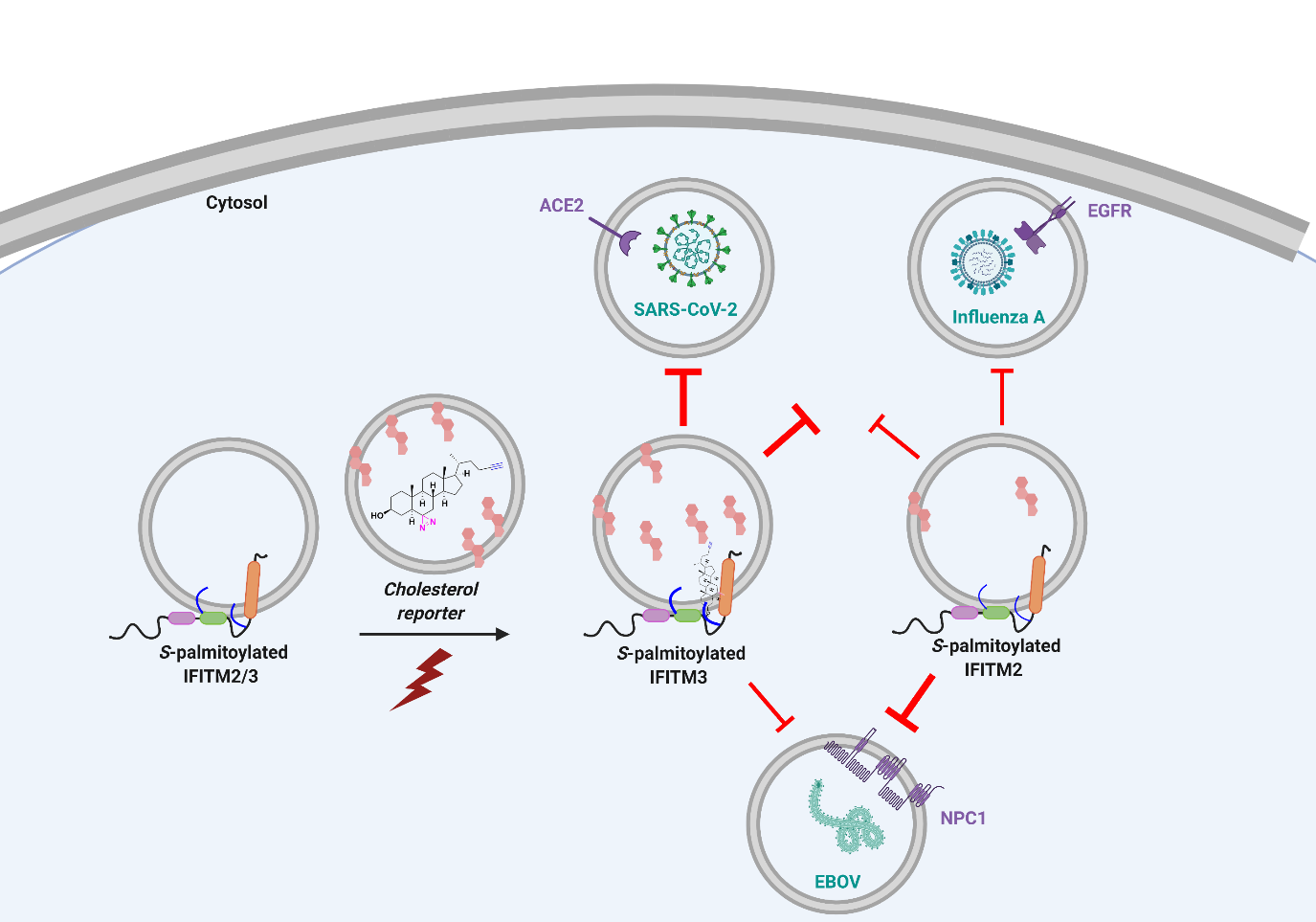

Supplement: Supplement 2 [file e8c5b5d3c6aaa9dd16801e84.png]
